# Comprehensive analysis of αβT-cell receptor repertoires reveals signatures of thymic selection

**DOI:** 10.3389/fimmu.2025.1605170

**Published:** 2025-09-19

**Authors:** Daniil V. Luppov, Elizaveta K. Vlasova, Dmitry M. Chudakov, Mikhail Shugay

**Affiliations:** ^1^ Institute of Translational Medicine, Pirogov Russian National Research Medical University, Moscow, Russia; ^2^ Institute of Personalized Oncology, I.M. Sechenov First Moscow State Medical University, Moscow, Russia; ^3^ Department of Biological and Medical Physics, Moscow Institute of Physics and Technology, Dolgoprudny, Russia; ^4^ Department of Information Technologies and Programming, Saint-Petersburg, Russia; ^5^ Center of Molecular Medicine, Central European Institute of Technology (CEITEC), Masaryk University, Brno, Czechia; ^6^ Department of Genomics of Adaptive Immunity Immunity, Shemyakin-Ovchinnikov Institute of Bioorganic Chemistry, Moscow, Russia; ^7^ Center for Molecular and Cellular Biology, Moscow, Russia; ^8^ Abu Dhabi Stem Cells Center, Abu Dhabi, United Arab Emirates

**Keywords:** thymic selection, immune repertoire sequencing, immune repertoire analysis, T-cell immunity, T-cell receptor repertoire, HLA alleles

## Abstract

Thymic selection is crucial for forming a pool of T-cells that can efficiently discriminate self from non-self using their T-cell receptors (TCRs) to develop adaptive immunity. In the present study we analyzed how a diverse set of physicochemical and sequence features of a TCR can affect the chances of successfully passing the selection. On a global scale we identified differences in selection probabilities based on CDR3 loop length, hydrophobicity, and residue sizes depending on variable genes and TCR chain context. We also observed a substantial decrease in N-glycosylation sites and other short sequence motifs for both alpha and beta chains. At the local scale we used dedicated statistical and machine learning methods coupled with a probabilistic model of the V(D)J rearrangement process to infer patterns in the CDR3 region that are either enriched or depleted during the course of selection. While the abundance of patterns containing poly-Glycines can improve CDR3 flexibility in selected TCRs, the “holes” in the TCR repertoire induced by negative selection can be related to Arginines in the (N)-Diversity (D)-N-region (NDN) region. Corresponding patterns were stored by us in a database available online. We demonstrated how TCR sequence composition affects lineage commitment during thymic selection. Structural modeling reveals that TCRs with “flat” and “bulged” CDR3 loops are more likely to commit T-cells to the CD4+ and CD8+ lineage respectively. Finally, we highlighted the effect of an individual MHC haplotype on the selection process, suggesting that those “holes” can be donor-specific. Our results can be further applied to identify potentially self-reactive TCRs in donor repertoires and aid in TCR selection for immunotherapies.

## Graphical Abstract

Study overview. **(A)** Datasets used in the study and the way TCR sequences were analyzed. **(B)** Main directions in which thymic selection shapes TCR repertoire. Selection results in TCR-CDR3 losing positively charged and large amino-acids while increasing its flexibility. Unlike CDR3 of the TCRα chain, CDR3β hydrophobicity is also increased by selection. CDR3s carrying Cysteines and glycosylation sites are unlikely to pass the selection. In contrast, CDR3s carrying poly-Glycine regions are more likely to be selected for both chains. T-cells committed to CD8+ lineage were more likely to feature bulged CDR3s compared to CD4 +. **(C)** CDR3s enriched after thymic selection guide lineage commitment according to single-cell RNA sequencing data analysis, e.g. the MAIT cells and CD8+ phenotypes. **(D)** Enrichment and depletion of certain TCR motifs pinpoint the fine structure of post-selection repertoires, as inferred by sequence neighborhood analysis. **(E)** Comparative analysis of enriched and depleted TCR clusters after thymic selection in monozygotic twins revealed that the selection process is shaped by HLA haplotype.

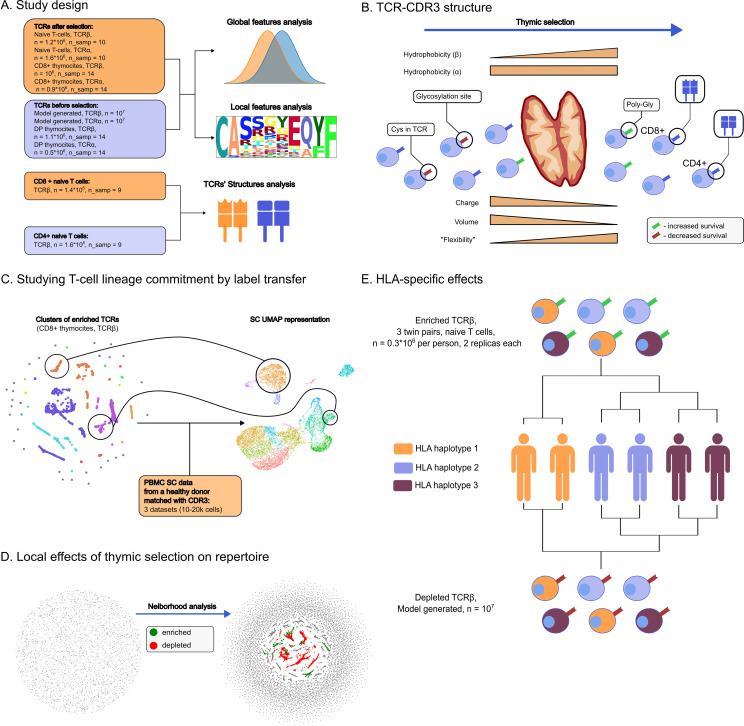

## Introduction

Lymphoid progenitors migrate from the bone marrow to the thymus where they develop T-cell receptors (TCR, a heterodimer of α and β chains) via a process called Variable-(Diversity)-Joining gene rearrangement, which undergo selection in order to become mature T-cells (1).

The process of V(D)J gene rearrangement involves two main steps: 1) three (V, D, and J for TCR β chain) or two (V and J for TCR α chain) gene alleles from corresponding loci are selected and recombined together; and 2) bases are randomly deleted at gene ends and non-template (N) nucleotides are added to the junction sites between genes to increase sequence diversity. These processes result in a great variety of possible TCRs, estimated to be ~10^19^ (2), which is orders of magnitude greater than the total number of T-cells in the human body (~10^11^ (3),).

Before release into the bloodstream, a T-cell must undergo thymic selection to ensure functionality in antigen recognition (positive selection) and tolerance to healthy cells, avoiding reactivity to self (negative selection) (1). This is a multi-staged process (4) that begins with CD4-CD8- double-negative (DN) T-cells rearranging their TCR β chain and validating their functionality with pre-TCRα. Next, they rearrange the α chain, forming a mature TCR, and transition to a CD4+CD8+ double-positive (DP) phenotype, undergoing positive and negative selection. Finally, DP T-cells differentiate into CD4+ or CD8+ single-positive (SP) T-cells and emigrate from the thymus.

The complementarity-determining region 3 (CDR3) of the T-cell receptor (TCR), encoded by the V(D)J junction and directly interacting with the peptide presented by MHC, is highly diverse and often serves as a proxy for TCR sequences in studies (5). This study focuses on CDR3 sequences to explore the thymic selection process within the TCR repertoire.

Modern TCR repertoire studies use high-throughput sequencing (Rep-Seq (6) or AIRR-seq (7) to analyze millions of TCR sequences from various biological samples. Given the limited published data on human pre-selection thymocyte repertoires, we and others have utilized a theoretical model of V(D)J rearrangement to model such repertoires (8). We also rely on the hypothesis that “singleton” T-cell clonotypes supported by a single mRNA molecule primarily represent naive T-cells to model the post-selection naive TCR repertoire (9).

Our general approach in the present study was to compare TCR repertoires before and after selection using model data and conventional peripheral blood mononuclear cell (PBMC) AIRR-seq data and to validate our findings using sorted DP and SP thymocytes (10). We explored both local and global repertoire structures of pre- and post-selection TCRs by analyzing sequence features that can influence the selection process on various levels: V/J gene usage, amino acid composition and physicochemical properties of primary CDR3 sequences, CDR3 k-mer profiles of repertoires, and prominent sequence motifs. In order to refine comparative analysis and remove noise originating from the intrinsic randomness of the V(D)J rearrangement, we utilized a TCR sequence cluster enrichment strategy based on the TCRNET method, allowing us to detect selection motifs in whole-body TCR repertoires with intrinsically complex structures (11).

Previous studies have examined TCR features in thymic selection. Lu et al. (12) reported shifts in amino acid usage, with reduced frequencies of hydrophobic and positively charged amino acids and cysteines. Stadinski et al. (13) found that TCRs with hydrophobic residues in specific positions are prone to cross-reactivity, reducing their survival chances. Other studies showed repertoire differences across the thymus, lymph nodes, and spleen (14, 15). These features were also revisited in Isacchini et al. (16) using repertoire modeling, yet the study arrives at the conclusion that selection features resemble themselves on local and global scales, claiming that there are no forbidden TCR sequences and selection motifs. We review the aforementioned features using our framework and arrive at similar conclusions, yet we extend previously published findings by showing locally enriched and depleted TCR patterns after selection that can be both public and donor/HLA-specific.

Linkage between lineage commitment and TCR was extensively studied, revealing distinct CDR3 features between CD4+ and CD8+ (17), CXCR3+ and CXCR3- naive CD8+ (18), and helper T cell subsets (19). Here we explore it in more detail via single-cell data, showing that selection motifs are linked to certain phenotypes. Moreover, we show that the structure of the CDR3 loop is different for CD4- and CD8-related TCR motifs.

We also considered the role of donor HLA haplotype in selection and demonstrated how allele-specific differences contribute to local repertoire characteristics. Such effects were previously reported for mice CD4+ repertoires (20). It was also shown that MHC context generally shapes the T-cell repertoire (21). An approach, similar to ours, involving twins’ TCR repertoires, was used by Tanno et al. to reveal the impact of genetic factors, in particular MHC alleles, on TCR repertoires (22).

## Materials and methods

### Post-selection T-cell repertoires

We used previously published (PBMC) TCR repertoire sequencing data for both TCRα (23) and TCRβ (24) chains. For TCRβ, a sample of 10 CMV donor repertoires selected *ad hoc* were chosen from the HIP cohort of the Emerson et al. (24) dataset for TCRβ analysis (sample IDs in dataset from 1 to 10). For TCRα we used all bulk TCRα PBMC data (10 samples) available in Heikkilä et al. (23). Only clonotypes that are supported by a single read (singletons) were used in subsequent analysis (9). The TCRβ sample consisted of 1,147,250 TCR sequences and the TCRα sample of 1,582,774 (**Supplementary Table 1**). Note that generation biases were not controlled, as the datasets comprised sequences from multiple individuals, which masked the effects of individual generation biases. Additionally, generation biases are imprinted in the selection process [see reference (16)].

### Sorted repertoires

Repertoires of sorted CD4+ and CD8+ naive (post-selection) T-cell sequencing were taken from Qi et al. (25); this data is available for TCRβ chain sequencing only. TCR repertoire DP and CD8+ (SP) thymocytes (pre-selection) were taken from a recent Quiniou et al. (10) study. Naive repertoires of all nine donors in this dataset were combined together, resulting in 1,346,776 clones for CD8+ naive cells and 1,599,217 clones for CD4+ naive cells (**Supplementary Table 1**). Note that for these datasets no read count information was used (i.e. all clonotypes were assumed to be singletons) in order to avoid potential amplification biases and to make it compatible with other datasets.

### HLA matched and mismatched repertoires (twin studies)

A dataset containing PBMC TCRβ repertoire sequencing for three pairs of monozygotic twins was taken from “Precise tracking of vaccine-responding T cell clones reveals convergent and personalized response in identical twins” by Pogorelyy et al. (26). Pre-vaccination and day 0 repertoires sampled prior to treatment were used as biological replicates for each twin, and only singletons were included in the analysis. HLA alleles for donors are listed in **Supplementary Table 2**. An additional dataset containing PBMC TCRα and β repertoires from three pairs of monozygotic twins was taken from “Distinctive properties of identical twins’ TCR repertoires revealed by high-throughput sequencing” by Zvyagin et al. (27). Sorted naive CD4+ T-cells TCRβ repertoires from two pairs of monozygotic twins were also obtained from “Functionally specialized human CD4+ T-cell subsets express physicochemically distinct TCRs” by Kasatskaya et al. (19). Summary statistics for datasets are reported in **Supplementary Tables 1**, **3**, and **4**.

### Simulating pre-selection V(D)J rearrangements

Pre-selection TCRα and β repertoires were simulated based on a theoretical probabilistic model of the V(D)J rearrangement process using OLGA software (v1.2.4) as described previously (8). The software was executed with default runtime parameters and model probabilities, random seed was set to 100, and a sample of 10^7^ random rearrangements was generated for each TCR chain.

### Single-cell data analysis

Single-cell datasets with assigned cell types totaling 178307 cells from PBMC samples of 88 healthy patients were taken from Lindeboom et al. (28). Cell type annotation was performed using CellTypist, as specified in the original study (29). Cluster abundance on a particular cell type was tested using the Fisher exact test.

### TCR amino acid sequence feature and motif analysis

Basic features of the TCR sequence such as V/J gene usage, single amino acid frequencies, k-mer (k=3) frequencies, and physicochemical properties of CDR3 regions were carried out using in-house scripts as described previously (30). The set of k-mers was not filtered based on their relative position in CDR3. Kidera factors (key amino acid features that describe most variance in polypeptide physicochemical properties) (31), charge, and hydrophobicity were calculated using the “peptides” python package (v0.3.2). The most informative Kidera factors, Kideras 2, 4, 6, and 8, correspond to Side-chain size, Hydrophobicity, Partial specific volume, and Occurrence in the alpha region respectively. Kidera factor values were compared using T-test; Cohen’s d was used as an effect size estimate.

Kidera factors were not z-score normalized because the datasets compared with OLGA-generated data were obtained using the same protocol as the data used to train the OLGA model (**Supplementary Table 1**) and all samples in the thymocytes dataset were obtained in a single batch. Detection of TCR CDR3 sequence motifs was performed using the TCRNET algorithm implemented in VDJtools (v1.2.1) as described previously (11). This method defines TCR sequences of interest as those that are placed in the more dense regions of a CDR3 sequence similarity graph compared to a control (typically produced assuming V(D)J rearrangement model with no selection pressure) dataset: the number of 1-hamming distance neighbors is compared to the expected number of neighbors adjusted for sample and control sizes to produce an enrichment score and a P-value based on Binomial approximation.

Note that in order to infer TCR sequence clusters that were depleted by negative thymic selection, we simply swapped “background” (control) and “foreground” (our sample of interest), i.e. we searched for TCRs enriched in pre-selection data compared to post-selection.

In order to produce a representative set of TCR clusters, we selected the top 10,000 neighbor-enriched CDR3 sequences based on enrichment P-value. Selected sequences were clustered by choosing connected components of the graph with edges connecting sequences that differ by a single amino acid substitution. Motifs for selected clusters were visualized using logomaker package (v0.8). The top five largest clusters were subsequently analyzed and numbered according to their cluster size rank. The number of clusters was selected *ad-hoc*.

The SoNNia (v0.2.3) model was additionally used to assess differences in amino acids’ occurrence probabilities, in particular their position in post- vs pre- selection repertoires (32). For calculating marginal probabilities of sequence features, we trained the SoNNia model using pairs of post-selection and pre-selection datasets (e.g. post-selection TCRβ and OLGA generated TCRβ data). Post-selection datasets were used as data for model inference and pre-selection datasets were used as data sampled from generative distribution. Each dataset was processed with methods from the “Processing” class and then passed to the SoNNia model with appropriate “pgen_model” parameters. Each model was trained for 50 epochs, with a batch size equal to 10^4^. Models were assessed with built-in plotting functions, as shown in the SoNNia tutorial (https://sonnia.readthedocs.io/en/latest/sonnia_tutorial.html).

### Comparative analysis of twins dataset

In order to identify positively and negatively selected CDR3 clusters in the twins dataset, we subsampled each twin sample to 306,553 CDR3s (size of the smallest repertoire) and pooled all the samples together. We used *ad hoc* thresholds to select significantly enriched (log2 fold change > 2 and -log10 p > 12 for sample pool compared to simulated sequences as control) and depleted (log2 fold change > 1 and -log10 p > 12 for simulated sequences compared to sample pool as control) clusters after thymic selection clusters. Clusters containing more than 10 sequences were used for further analysis. Next, similarity between positively and negatively selected clusters was estimated by computing the Jensen-Shannon divergence between cluster frequencies defined as the number of clonotypes from a given cluster present in a given sample.

### Structure analysis

CDR3 loop structures for TCRs of interest were modeled using the TCRmodel web tool (33) and processed with Pymol (version 2.3.0). Our in-house “mir” software package was used to annotate the resulting PDB files (see (34)). As CDR3α is not known in most of our datasets, we used a generic CAGGSSNTGKLIF (TRAV27, TRAJ37) sequence that was the most commonly observed variant in the dataset from Heikkila et al. (23) as a dummy TCRα sequence. The TCR CDR3 backbone was visualized by applying PCA to Cα atom coordinates.

Experimental structures of TCR:pMHC complexes were obtained from VDJdb (35). We selected only records with available PDB IDs of human TCRs. The structures were required to harbor both TCRα and TCRβ along with a pMHC complex. Additionally, at least one CDR3β residue had to be within 5 Å of the peptide in a pMHC complex to ensure direct contact between the TCR β chain and the pMHC complex. A total of 154 structures in total were analyzed. Dihedral angles in these structures were calculated with Biopython (v. 1.85) Python package (36).

### Code availability

All code used in this study is available at https://github.com/LuppovDaniil/Thymic_selection_notebooks (Python version 3.11.5., R version 4.1.2).

## Results

### Comparing pre- and post-selection TCR amino acid sequences

AIRR-seq data for DP T-cells sorted from the thymus can be used to explore the initial space of rearranged TCRα and β sequences existing prior to positive and negative selection, similar to the work of Quiniou et al. (10). Recent studies demonstrate that a probabilistic model can accurately replicate the structure of V(D)J rearrangement space, generating TCR sequences with amino acid composition and frequencies resembling those produced *in vivo* (8). Here we use both Quiniou et al. and model datasets as a pre-selection repertoire.

There are several ways to acquire the TCR repertoires of post-selection T-cells that have not yet undergone strong antigen exposure and obtained memory phenotypes. One can either sort and sequence naive CD4+ and CD8+ T-cells as performed in Qi et al. (25) or use SP thymocytes as done in Quiniou et al. (10). Alternatively, one can select T-cell clonotypes detected only once (singletons) from unsorted PBMC AIRR-seq data, as they mostly represent naive T-cells (see *Britanova et al.* (9)). In the present study, we used the datasets from Qi et al. and Quiniou et al. and selected singletons from 10 samples chosen *ad hoc* from the Emerson et al. dataset as a TCR repertoire after selection but prior to any antigen exposure or subsequent expansion. The detailed description of these datasets and comparison analysis is given in the Method section.

**Figure 1:**
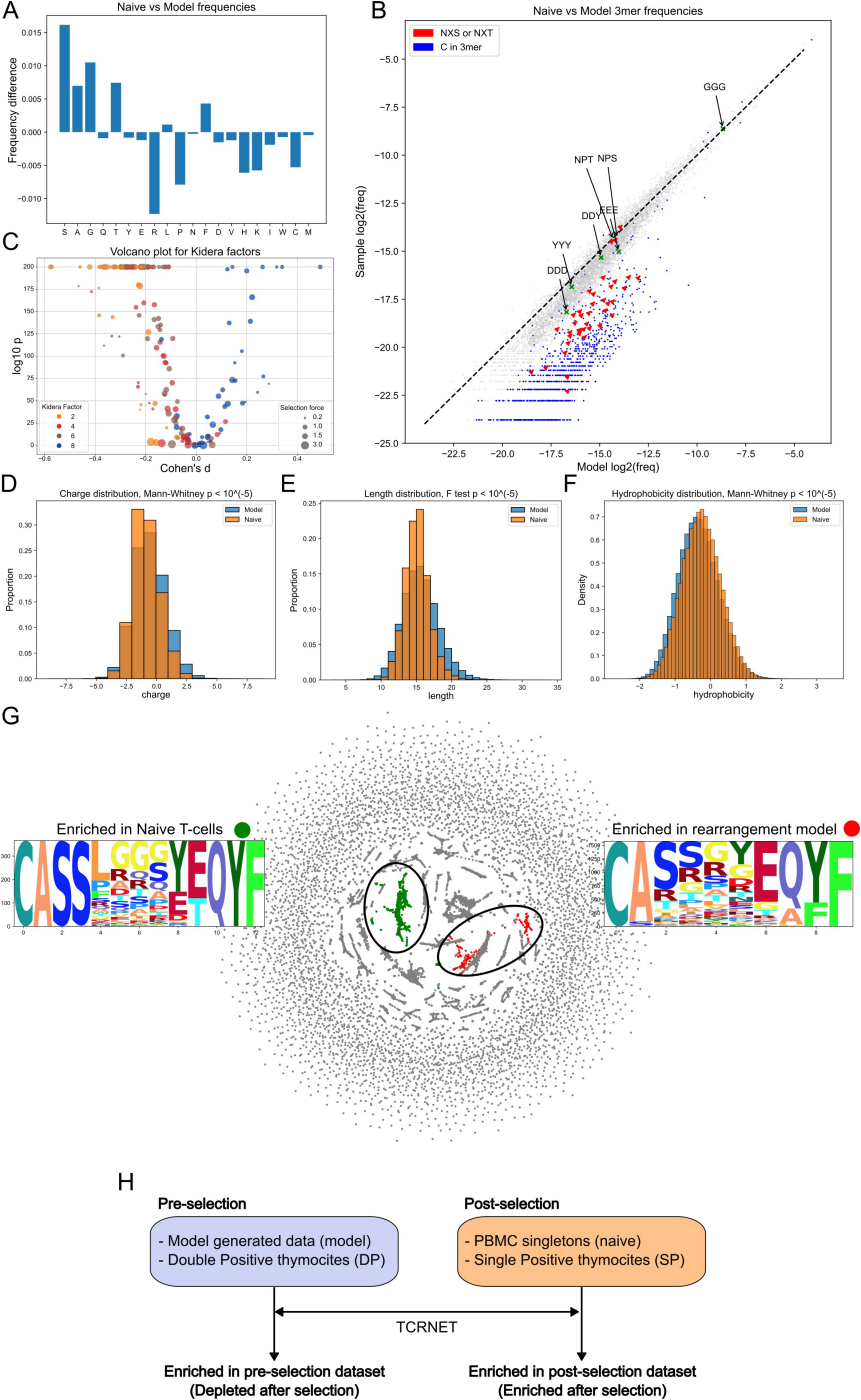
Model generated CDR3β and naive CDR3β repertoire (naive sample) comparison. **(A)** Single nucleotide frequencies before and after selection comparison. **(B)** 3-mers frequencies comparison. Glycosylation sites (red) and C-containing 3-mers (blue) are negatively selected. Dashed line represents the y = x relationship. **(C)** Volcano plot for Kidera factors affected by selection. Each point represents a VJ pair. Negative shift in Kidera factors 2, 4, and 6 and positive shift in Kidera factor 8. The ratio of fractions of a particular VJ pair before and after selection were labeled as “selection force”. **(D)** Negative shift for charge distribution after selection. **(E)** “Winsorizing” of CDR3 length values post selection. **(F)** Positive shift in hydrophobicity values after selection. **(G)** Largest enriched and depleted clusters and their logos obtained by sequence neighborhood analysis. **(H)** Study nomenclature for the comparison groups. Datasets utilized as repertoires before thymic selection (pre-selection) are shown on the left side of the figure and the ones after selection (post-selection) on the right. Arrows represent enrichment analysis (TCRNET) application with pre- or post- selection dataset taken as background and the relative dataset from the opposite group taken as repertoire under examination. Sequences enriched in post-selection datasets are also referred to as enriched after selection. Sequences enriched in pre-selection datasets - as depleted after selection.

Analysis of TCRβ CDR3 amino acid frequencies revealed a significant post-selection decrease in specific residues compared to those expected from a V(D)J rearrangement model (**Figure 1A**). Positively charged and physically large amino acids, such as arginine, histidine, and lysine, were likely reduced due to strong antigen binding or steric hindrance in antigen recognition (37), while Proline and Cysteine were negatively selected possibly due to their effects on TCR structure. These findings were generally confirmed in thymocyte data (**Supplementary Figure 1A**).

Analysis of 3-mer frequencies revealed that 3-mers with cysteine had less chance to survive selection (**Figure 1B**), consistent with observations at the single amino acid level (**Figure 1A**). 3-mers with the NX[S,T] motif, associated with N-glycosylation sites (38), are less likely to survive selection, with NPT and NPS being the least affected, consistent with the absence of glycosylation at these sites (39) (**Figure 1B**). We also analyzed sulfonation site motifs, which were described in Pospelova et al. work (40) for antibodies, such as DDD, DDY, YYY, and EEE. The tangible reduction effect was observed only for the DDD motif. Both glycosylation sites and Cysteine-linked effects and a lack of effect from putative sulfation sites were observed in the thymocytes data (**Supplementary Figure 1B**). Additionally, we assessed the positions of the glycosylation sites in the CDR3 sequences and discovered that, for CDR3β, most (85% and more for different datasets) glycosylation sites were located in the NDN segment, highlighting their somatic origin. For CDR3α, however, only 44-53% of these sites were located in the N segment.

In order to describe changes in physicochemical properties of TCRβ CDR3s, we harnessed Kidera factors (31). These factors represent the key physical properties of amino acids obtained by dimensionality reduction. We compared each VJ pair independently in order to lessen the bias caused by the choice of the V and J genes (**Figure 1C**). The Kidera factors negatively affected by selection were Kidera 2, which determines side chain size, Kidera 4, which is inversely associated with hydrophobicity, and Kidera 6, which determines partial specific volume. These results suggest that TCRβs with a physically small hydrophobic CDR3 have a greater chance of passing selection. The only factor that increased post-selection was Kidera 8 (occurrence in the α-helix structural region). Notably, the selection effect within a particular Kidera Factor was shared across all VJ pairs. Identical analysis of DP and SP thymocytes mirrored the above results, however, the effect for Kidera factor 6 was less pronounced (**Supplementary Figure 1C**).

The analysis of the physical properties of TCRβ CDR3 revealed a decrease in repertoire charge post-selection (**Figure 1D**), consistent with the observed reduction in frequencies of positively charged amino acids (**Figure 1A**), the winsorizing (resulting distribution having thinner tails) of lengths (too short and too long sequences both have less chance to pass selection), and the increase in hydrophobicity (**Figure 1F**). Except for length, which experienced shortening instead of winsorizing, the same effects were detected on thymocytes data (**Supplementary Figures 1D–F**).

Next, we identified functional clusters of TCRβ CDR3s enriched in naive cells compared to the modeled sample and vice versa (30). The largest enriched clusters in naive and model-generated samples are presented in **Figure 1G**. Model-enriched clusters can be interpreted as sequences which tend not to pass the selection and naive sample-enriched clusters are ones which are likely to survive thymic selection. For convenience, the terminology (enriched and depleted pre- and post-selection versus positively and negatively selected) used here and in the **Supplementary Materials** is explained in **Figure 1H**.

The majority of the post-selection enriched clusters contain poly-Glycine sequences in the middle of a TCRβ CDR3 (**Figure 1G**; **Supplementary Figure 2**), which is known to be one of the most flexible among the polypeptides chains (41). Additionally, k-mers analysis demonstrated that GGG 3-mer passed through the selection with unchanged frequency (**Figure 1B**; **Supplementary Figure 1B**), confirming the above finding. CDR3β from pre-selection enriched clusters frequently contained Arginine and Proline (residues likely impacting CDR3 loop structure) and exhibited deviations in the CASS consensus sequence at the CDR3 start (**Figure 1G**; **Supplementary Figure 2**). Results of enriched cluster analysis for thymocytes data mostly resembled those obtained in the generated data (**Supplementary Figure 3**).

In order to obtain pre- and post- selection probabilities of amino acid occurrence in a particular position in CDR3, we utilized the SoNNia software (32). Generally, we observed the nearly identical to enriched clusters analysis trends (**Supplementary Figures 4A, С**).

Gene usage analysis revealed a vague picture of little or no preference of selection to more frequent genes (or “rich get richer effect” (42)) on both generated and experimental data (**Supplementary Figures 5, 6**).

Interestingly, while no “rich get richer effect” was observed for the gene frequencies, it was detected in terms of CDR3β generation probabilities (pgens) for DP and SP thymocytes calculated via the OLGA model (p < 10^-5^, Mann Whitney test, differences of median log2 *Pgen* between SP and DP was 2.24, **Supplementary Figure 1G**).

Analysis of TCRα CDR3s, conducted identically to the one for TCRβ, revealed both common selection effects and notable differences between the two chains (**Supplementary Figures 4, 7–12**). For this analysis, a subsample of CDR3 singletons from PBMC (23) was utilized alongside a thymocytes CDR3α repertoire (10) and model-generated data.

Among the observed differences, the following points are noteworthy: (i) there were no clear trends in the selection preferences toward individual amino acids (**Supplementary Figures 7A, 8A**) (ii) there was no consistent direction of Kidera factor changes for TCRα VJ pairs (**Supplementary Figures 7C, 8C**); (iii) there was an unchanged post-selection repertoire hydrophobicity (**Supplementary Figures 7F, 8F**); and (iv) there was a more complex structure of clusters enriched and depleted after selection, with DS and NY 2-mers prevalent in thymocyte-enriched clusters but not in naive data (**Supplementary Figure 10**) and SS 2-mers being more abundant in clusters enriched in both model and DP thymocytes (**Supplementary Figures 9, 10**). Notably, the common selection effects observed for both α and β chains were less pronounced in the case of TCRα (**Supplementary Figures 7, 8**).

Additionally, we stored TCRs and patterns for the largest clusters enriched and depleted after thymic selection, obtained above in a database available at https://github.com/LuppovDaniil/thymic_selection_motifs_database.

### Single-cell analysis reveals the CDR3-dependent differentiation

To trace the signs of TCR driven lineage commitment during thymic selection, we utilize single-cell sequencing PBMC data derived from 88 healthy donors, totaling 178307 T-cells of different lineages (28). We aimed to demonstrate that enriched and depleted clusters are linked with particular T-cell phenotypes. We intersected CDR3s from these clusters (**Supplementary Figures 2, 3, 9**, **10**) with a single-cell TCR repertoire and tested the linkage of these clusters with each particular cell phenotype.

**Figure 2:**
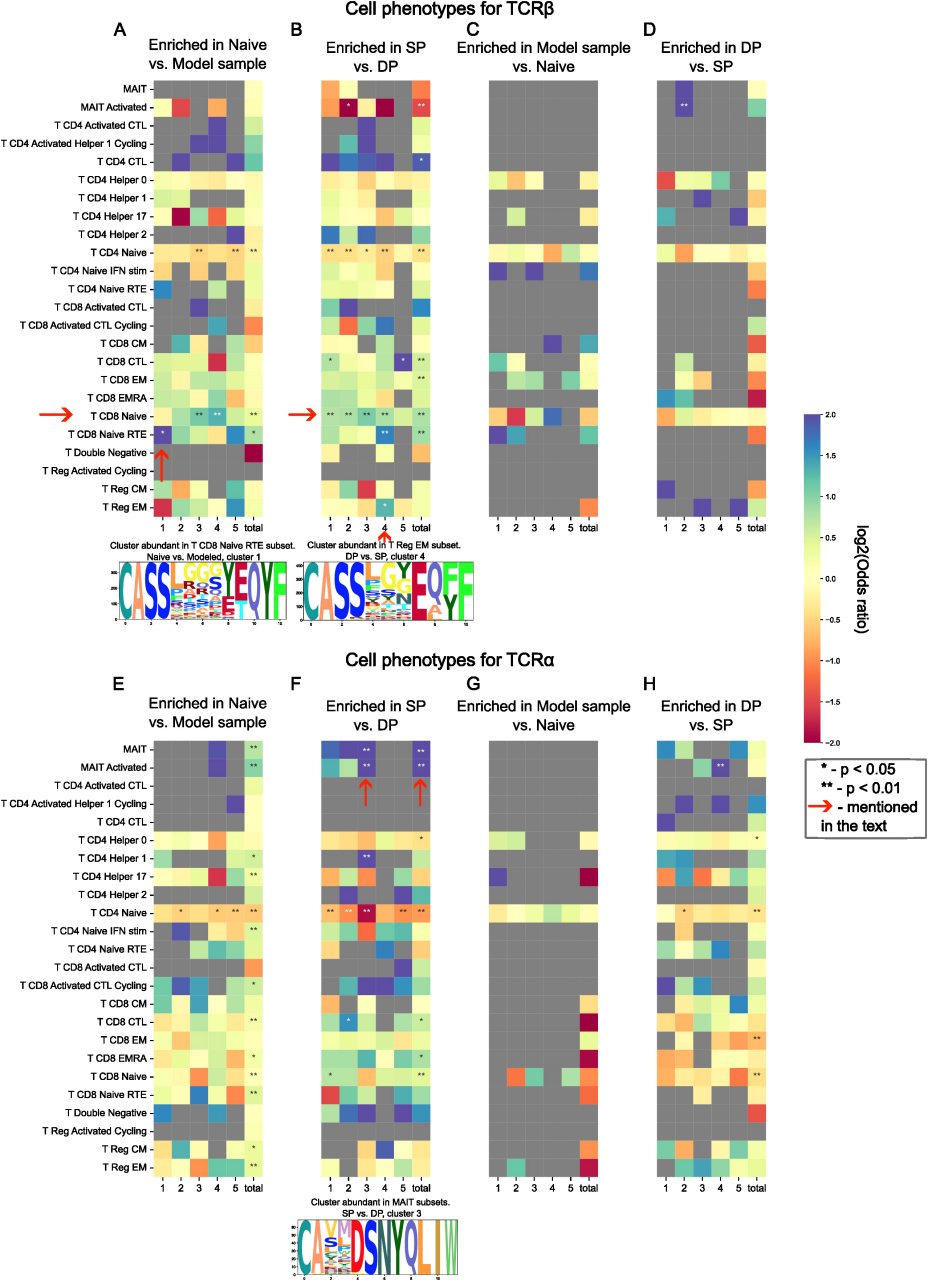
Abundance of TCR sequence motifs enriched and depleted during thymic selection in T-cell subsets defined by single-cell sequencing data. Odds ratio of observed to expected number of sequences matching between cell phenotype and selection “motif” (cluster) is shown by color; two-tailed Fisher exact test P-values for odds scores post multiple testing correction are shown with asterisks (* for p < 0.05 and ** for p < 0.01). Cluster-phenotype pairs discussed in the main text are highlighted with arrows. Chosen clusters are represented as logos. SP, DP, Naive, and Model datasets for TCR α and β are shown in panels **(A–H)** which represent different comparisons as described in the main text: **(A, B, E, F)** represent enriched after selection TCRs, while **(C, D, G, H)** represent depleted after selection ones; **(A, C, E, G)** are based on model data and naive cells, **(B, D, F, H)** are based on real data; **(A–D)** describe β chain, and **(E–H)** describe α chain.

Generally, the expected results were obtained. TCRs from clusters depleted after thymic selection were 10- to 20-fold less abundant in single-cell data than those from enriched clusters (**Figures 2C, D, G, H**), supporting our ability to identify TCRs disfavored by selection. Clusters enriched post-selection were mostly linked with naive phenotypes (**Figures 2A, B, E, F**). SP thymocyte clusters were mostly represented by CD8+ naive cells, which is expected since SP thymocytes in our research are of CD8+ lineage (**Figure 2B**).

We also found that the TCRβ of CD8+ cells are more prone to neighborhood enrichment than TCRβs of CD4+ cells (**Figure 2A**). The cluster enriched in CD8+ Recent Thymic Emigrants (RTE) subtype stood out against the other clusters enriched in CD8+ Naive cells (**Figure 2A**). Such an observation could be the sign of peripheral selection effects on the repertoire. Cluster 4 of SP thymocytes is also of particular interest, since it demonstrated abundance in both CD8+ RTE and Tregs (**Figure 2B**).

Moreover, we observed the abundance of TCRα from enriched SP thymocyte clusters in MAIT cells subtype (**Figure 2F** cluster 3), which is again expected since MAIT cells are characterized by their “semi-invariant” TCRα (43).

Additionally, we assessed our assumption considering mostly naive origins of singletons in PBMC data. We inferred the phenotype for singletons from TCRβ and TCRα datasets using single-cell data and found that TCRβ singletons were indeed enriched in the naive subset (p < 10^-4^, Fisher exact test); however, we were unable to detect the same enrichment for TCRα. Despite this peculiar inconsistency, we still believe that our assumption is correct, since the share of memory cells in the single-cell data was 10 times lower than the share of naive cells and the majority of T-cells were once naive.

To consolidate our findings, we analyzed SP thymocyte clusters utilizing standard 10X Genomics datasets containing both single-cell gene expression and TCR sequencing data. Generally, similar results were obtained: (i) cells carrying TCRβ from CD8+ thymocyte-enriched clusters were mostly of CD8+ phenotype; and (ii) TCRα-enriched clusters were mostly associated with MAIT cells (**Supplementary Note 1**).

These results demonstrate TCR driven lineage commitment during thymic selection both for a well-characterized case of MAIT cells as well as a more complex one of CD8+ cells.

### Structural analysis confirms CDR3 features of CD4+ and CD8+ T-cells

**Figure 3:**
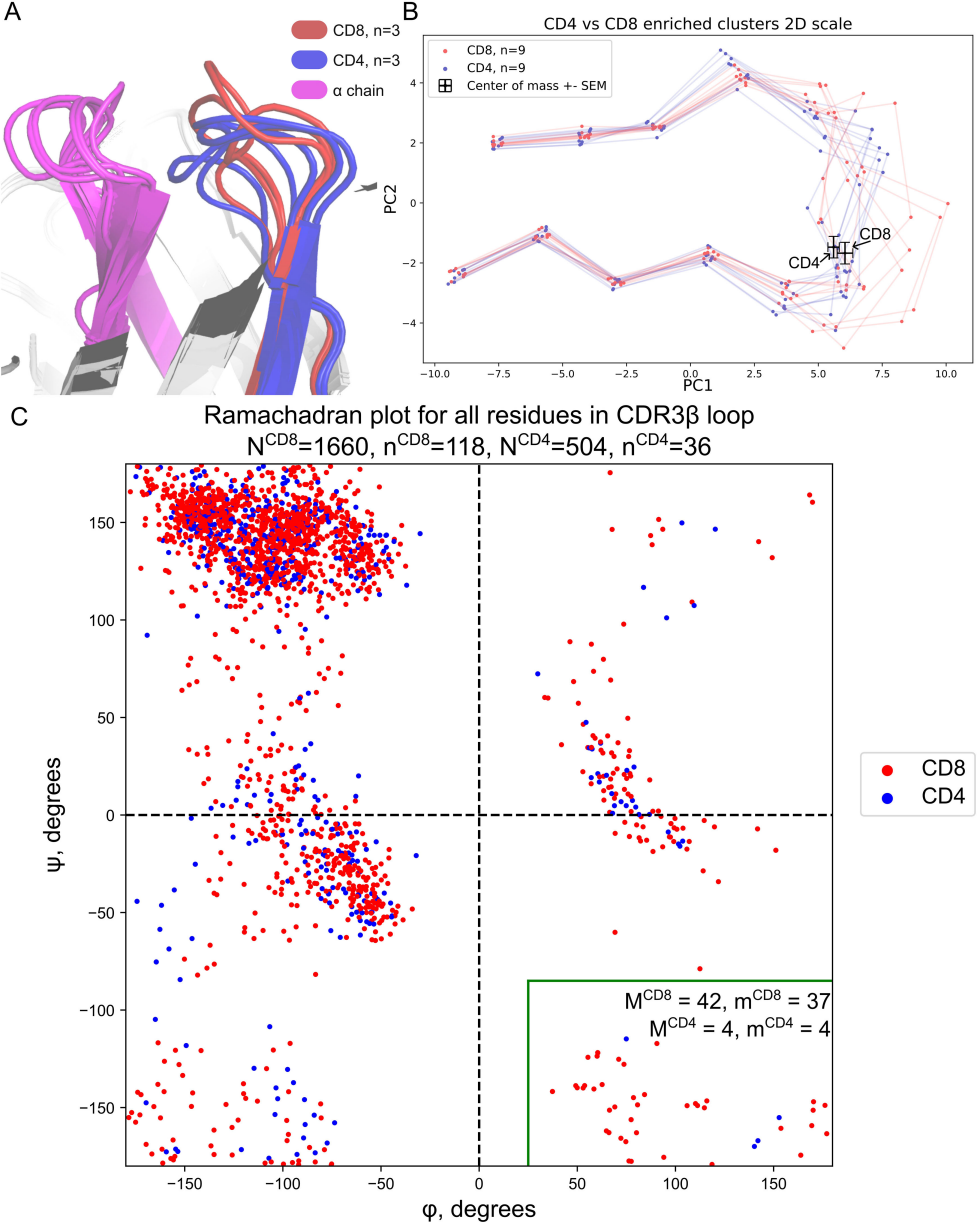
Structural differences between CDR3 of CD8+ and CD4+ T-cells. **(A)** Modeled 3D structures of CD4- and CD8-enriched CDR3β repertoires. The most common CDR3βs were taken from the three largest clusters for both CD4+ and CD8+ clones for visualization. CD8-enriched structures tend to be more loose while CD4-enriched clusters are more assembled. **(B)** Same as A but 2D PCA projection with two additional TCRs from each cluster. Centers of mass of the contacting part of CDR3 with error bars representing standard mean errors (SEMs) of PC1 and PC2 are shown with black crosses. **(C)** Ramachandran plot for CDR3β loops taken from known TCR:pMHC structures from VDJdb. The green rectangle highlights the CD8+-specific region of the plot. N^CD4/CD8^ represents the total number of residues taken from CD8+ or CD4+ TCRs respectively, n^CD4/CD8^ represents the total number of CD8+ or CD4+ structures respectively. Same but M and m are shown with numbers in the green rectangle.

Next, we compared CD4+ and CD8+ naive cell TCR repertoires taken from the Qi et al. study (25). The most notable result of this comparison is from clusters enriched in CD4+ or CD8+ CDR3βs. We employed the TCRmodel2 to analyze the structure of the most prevalent CDR3 in the largest clusters within each group (33) and discovered a difference in convexity between enriched CDR3β CD8+ and CDR3β CD4+ structures (**Figures 3A, B**). It appeared that CD4+ enriched clusters were structurally flat, whereas CD8+ clusters were more convex. The centers of mass for the contacting region of CD8+ CDR3β (excluding the first four and last five residues) were located further from the loop center compared to the center of mass of CD4+ CDR3β (**Figure 3B**). However, in our sample of 18 structures, this difference was not statistically significant.

This fact may be explained by the conformation of peptides in MHC class I and MHC class II grooves. MHC class I, which is recognized by CD8+ cells, tends to present peptides with a middle bulge, whereas MHC class II, recognized by CD4+ cells, tends to present flat peptides (44).

We further investigated the structural difference between CD4+ and CD8+ CDR3βs using data stored at VDJdb (35). We took Human TCRs with available TCR:pMHC complexes harboring contacts between CDR3β and peptide. In total 154 structures were analyzed. We visualized all CDR3β dihedral angles in these structures via a canonical Ramachandran plot and discovered a distinct region on the plot that was abundantly inhabited by CD8+ CDR3β residues (**Figure 3C**, green rectangle). The structural conformation corresponding to this region of the Ramachandran plot is mostly available for Glycines (45). The majority of structures with residues in this region were represented in it only by one residue. CD8+ TCRs harboring such a conformation were 2.83 times more frequent than CD4+ TCRs (p = 0.017, Fisher exact test).

This region at the right bottom of the Ramachandran plot in the Hollingsworth and Karplus work (45) was referred to as P_II_’. P_II_’ is viewed as a mirror region of P_II_ (more commonly referred to as Polyproline-II Helix), which is known to maximize polypeptide chain entropy and expose all hydrogen bond capable backbone atoms to the water. Additionally, this structural region frequently forms protein-binding motifs (46), which is relevant for TCR recognition of antigens. Thus, we would expect this CD8+ specific conformation to be relatively convex as we described it above. Moreover, the majority of residues in the discussed region were Glycines, which were in direct contact with the peptide. The data for the Ramachandran plot with corresponding PDB ID is available in **Supplementary Table 5**.

### HLA allele haplotype affects the selection

Next, we considered the HLA allele influence on the thymic selection. It is still a subject of debate whether HLA alleles affect the selection or not (16).

We used CDR3s bulk sequencing data of three pairs of twins aged 20 to 23 to address this question (26). This data contains TCRβ repertoires for three pairs of twins (assigned as S, P and Q) in two replicas: day of vaccination before the shot (day 0) and a day prior to vaccination day (pre-day).

We compared their naive CDR3 clusters to the modeled background repertoire. On the one hand, we expected their enriched naive CDR3 clusters to be closely related with each other since twins from the same pair have the same HLA alleles. On the other hand, we anticipated twins from different pairs to be different from each other in context of their naive TCR repertoire.

We extracted the same number of CDR3s from each twin sample and then clustered the enriched sequences from each sample together. Having done this, we obtained a number of clusters composed of sequences from different samples. Supposedly, each cluster contains CDR3s, which are functionally similar to each other (recognize similar peptides in the MHC-peptide complex). Thus, we would anticipate CDR3s derived from the relative pair of twins to fall into the same clusters.

**Figure 4:**
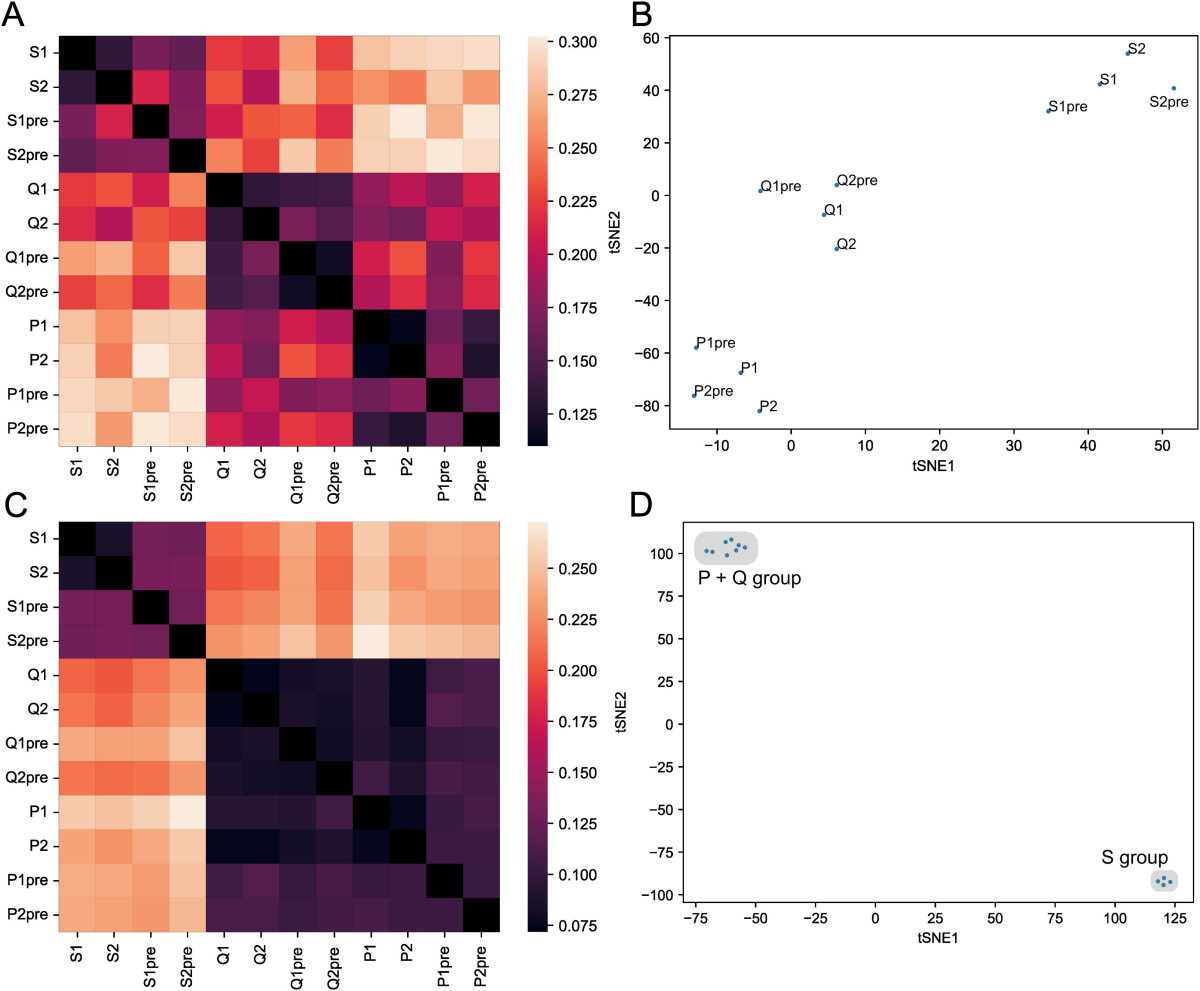
HLA allele affects thymic selection of the TCRβ chain. **(A)** Heatmap representing the Jensen-Shannon divergence between frequencies of occurrence in enriched functional clusters for each twin sample (*S*, *P*, and *Q* pairs in two replicas). **(B)** t-SNE plot for occurrence frequencies in positively selected functional clusters. **(C)** Heatmap representing the Jensen-Shannon divergence between frequencies of occurrence in modeled data functional clusters with each twin sample as a background. **(D)** t-SNE plot for occurrence frequencies in negatively selected functional clusters.

To verify this hypothesis, we analyzed the proportion of CDR3s from a particular twin in the clusters obtained. In this representation, each sample can be viewed as a discrete probability distribution of appearing in a particular cluster. This allowed us to calculate the Jensen–Shannon divergence between samples. We discovered that samples within each twin pair are much closer than samples from different twin pairs (**Figures 4A, B**).

Notably, twin pairs with the homozygous HLA-A 02:01 allele (pairs P and Q, please see **Supplementary Table 2**) appeared closer to each other than to the S pair, which had only one copy of HLA-A 02:01 allele.

The analysis of the clusters that were depleted during the selection showed similar results (**Figures 4C, D**). Thus, HLA alleles affect not only which TCRs will be promoted by selection but also the TCRs which will be eliminated by it, so called “holes” in the repertoire. Notably, we detected the effect of homo/heterozygosity of HLA-A gene alleles on the T-cell repertoire again but this time it was stronger—twin pairs, which were homozygous by HLA-A (P and Q), were 2–3 times closer to each other than to HLA-A heterozygous twin pairs (S) (**Figure 4D**).

We repeated the analysis in the same manner for two other sets of twins’ TCRs, one of which represented CD4+ naive cells, and obtained similar results (**Supplementary Note 2**).

These results suggest that one can observe the imprint of HLA-based selection on a naive repertoire. Moreover, this imprint was observed in both enriched and depleted clusters, indicating that particular HLA alleles can be favorable for one TCR and unfavorable for another.

## Discussion

In this study we sought to identify and investigate factors that are crucial for passing thymic selection. We considered such factors as amino acid composition, K-mers composition, Kidera factors, charge, length, hydrophobicity, and TCR gene usage. We also analyzed functional clusters enriched in samples before and after selection and SoNNia-inferred marginal probabilities of amino acids to occur in a particular position of CDR3. To gain a deeper understanding of enriched or depleted clusters after selection, analysis of physical structures and single-cell data analysis were carried out. Finally, we considered HLA alleles as a factor that has an impact on selection and indeed managed to show a significant influence from it.

Amino acid usage analysis revealed that having Proline and large positively charged amino acids in CDR3 reduces the likelihood of TCRβ but not TCRα survival during selection (**Figure 1A**; **Supplementary Figure 1A**). On a 3-mers level we showed that Cysteine and N-glycosylation sites negatively affected the chances of survival (**Figure 1B**; **Supplementary Figures 1B**, **7B**, **8B**). Kidera factors analysis revealed a difference in selection effects for α and β CDR3s. For CDR3β, we observed synchronous changes in four Kidera factors for every VJ pair (**Figure 1C**). These four Kidera factors represent physical volume, hydrophobicity, and occurrence in the alpha region. Thus, small, hydrophobic, and Glycine-reach CDR3βs have greater chances of surviving selection. Notably, the preference of the selection toward glycines was previously shown by Elhanati et al. (42). Unlike CDR3β, we did not observe the common direction of changes for the CDR3α Kidera Factors for every VJ pair (**Figure 1C**; **Supplementary Figures 7C, 8C**). This may indicate that the selection of physical properties in the case of CDR3α is specific to the VJ pair. We observed a reduction in the charge and length of CDR3α and CDR3β repertoires following selection (**Figure 1D**; **Supplementary Figures 1E, 7D, 8E**), with hydrophobic CDR3β sequences exhibiting higher survival probability (**Figure 1F**), whereas hydrophobicity did not influence CDR3α selection (**Supplementary Figures 7F, 8F**), likely due to possible VJ-specific selection factors. Furthermore, CDR3 carrying flexible poly-Glycine subsequences tend to survive the selection, while structurally complex ones exhibit the opposite (**Supplementary Figures 2, 3, 9, 10**). Gene usage analysis demonstrated a vague picture of gene usage changes through the selection (**Supplementary Figures 5, 6, 11, 12**).

Previous studies assessing the incidence of glycosylation sites in antigen receptors were limited to antibodies, highlighting *de-novo* acquisition of glycosylation sites as a distinctive property of follicular lymphoma (47): levels of N-glycosylation acquisition differ between follicular lymphoma subtypes and may have an important role in this malignancy (48, 49). Therefore, we might propose that acquired N-glycosylation may have a diagnostic value in case of T-cell abnormalities.

Overall, our analysis showed that structurally simple and flexible CDR3s have a greater chance of passing the selection. One can hypothesize that such CDR3s are capable of recognizing a wide variety of peptides within the thymus with a moderate binding strength, which is the exact requirement for getting through thymic selections (50) and is in line with the existing model of TCR recognition of a pMHC complex (51–53). The observed selection bias toward flexible TCRs aligns with the model where TCR changes its conformation to “scan” each pMHC complex (51–53). Small and hydrophobic TCRs may be better at binding and stabilizing pMHC complexes due to sterical and kinetic reasons: it was shown that both association and dissociation of the TCR:pMHC complex requires overcoming high energetic barriers and the above features may help to overcome such barriers (53).

Notably, observed differences in selection preferences for CDR3α and CDR3β remain enigmatic. The most obvious explanation lies in the fact that β chains contain the diversity region that typically translates to poly-Glycines, leading to an inherent bias. The presence of non-conventional T-cells in our analysis, such as MAIT and iNKT cells, which are selected by alternative mechanisms mostly driven by α chain (43), may partially explain this contradiction. Another possible explanation for these inconsistencies may be in the presence of a rescue mechanism for the α chain; unlike TCRβ, TCRα can undergo rearrangement multiple times during functional TCR formation (1). Thus, it may play a compensatory role for suboptimal TCRβ chains.

In addition to the above findings, we demonstrated the direct impact of TCRs on T-cell lineage commitment (**Figure 2**) using single-cell datasets with adjusted CDR3 sequencing data. The effect was especially pronounced for the α chain. TCRα plays a key role in commitment to non-conventional lineages like MAIT cells, which were highly enriched in our analysis (**Figure 2**; **Supplementary Note 1**) (43). Moreover, we discovered that flat CDR3s favor CD4 lineage, as they align with MHC class II peptides, while curved CDR3s are associated with CD8+ T-cells, resembling MHC class I peptides (44) (**Figure 3**). Broader analysis of available TCR:pMHC structures allowed us to identify CD8+-specific CDR3 conformations on the Ramachandran plot. Further analysis in this direction may aid in developing machine learning methods for T-cell fate prediction based on CDR3 sequences.

Finally, we demonstrated the effect of HLA alleles on thymic selection using CDR3 repertoires from three pairs of twins.

The presence of a HLA allele-mediated effect on thymic selection observed in our study contradicts prior findings (16), which suggested an overall lack of forbidden sequences for the selection, thus neglecting the HLA-alleles’ impact on the process of thymic selection. We suggest that this discrepancy can be explained by methodological differences: Isacchini et al. concentrated their effort around the repertoire as a whole, averaging local differences across repertoires at different TCR nearest neighbor graph scales, thus paying little attention to individual rare clusters specific to each sample from each subject being analyzed. For example, shortening of CDR3 sequences and removal of rare lengths will increase the number of nearest neighbors calculated using Hamming distance, trivially explaining the observation that “local properties of individual repertoires are well captured by the model and that the probability landscape of finding receptors sequences is relatively smooth as a function of sequence distance” reported by the authors. General trends, e.g. the Matthew effect for generation probabilities, can be detected by such analysis, exactly as shown in the paper under discussion. However, as discussed in the ‘HLA Allele Haplotype Affects the Selection’ section, only upon close examination of the composition of local patterns does it become clear that HLA-driven donor-specific negative selection operates in the space of functional TCR clusters. One can argue that monozygotic twins may carry the same initial recombination biases, leading to the proximity of repertoires. However, the authors of the aforementioned study have previously shown that the V(D)J rearrangement model depends little on genetic background; neither does the argument explain the fact that two pairs of twins with homozygous HLA-A 02:01 alleles were significantly closer to each other compared to a pair of twins with a heterozygous HLA-A gene (**Figure 4**). So, while the impact from VDJ recombination biases is strong, the overall selection is not driven exclusively by them. Also, Isacchini et al. analyzed the general landscape of the selection process by applying dimensionality reduction to trained selection models. We speculate that these generalizations in the analysis may lead to the conclusion that there are no forbidden and favored-by-the-selection motifs: the simplest example of such forbidden patterns to consider are glycosylation sites which can occur in TCR sequences with the high probability of generation but at the same time prevent them from passing the selection. Interestingly, previous studies in mice also demonstrate that CD4+ repertoires from animals homozygous by HLA is more diverse than the repertoire from heterozygous ones (54). Additionally, in a recent study, which involved a large cohort of 1,521 COVID-19 subjects, the strong HLA-TCR repertoire interplay was demonstrated (55).

The effect of HLA haplotype on thymic selection described here adds another level of complexity to the understanding of the number of autoimmune diseases where HLA risk alleles were identified (e.g Type I Diabetes (56) or Multiple Sclerosis (57)). We can suggest that the particular HLA risk alleles not only show immunogenic peptides to T-cells but also shape the repertoire itself, allowing autoimmune T-cell clones to survive the selection by promoting or at least not eliminating them.

Overall, our findings have a number of applications, including the accession of the likelihood of the particular TCR to pass the selection. It could be helpful in studying autoimmunity and for the development of future machine learning models for “in-silico thymic selection”. In immunotherapy, these results could aid adoptive T-cell transfer (58) by selecting TCR variants that mimic natural ones, improving antigen affinity and reducing side effects. Additionally, glycosylation sites should be avoided in the design of chimeric antigen receptors, immune checkpoint inhibitors, and any other therapeutic antibodies. All things considered, our research identified a number of TCR characteristics that significantly influence thymic selection. These results offer a quantitative explanation of the entire selection process and a clearer picture of the range of potential repertoire feature changes that may take place during thymic selection. It also provides an avenue for additional study and experimental validation, along with potential real-world applications.

## Data Availability

The original contributions presented in the study are included in the article/**Supplementary Material**. Further inquiries can be directed to the corresponding author. Database of enriched and depleted post-selection CDR3 clusters is stored at https://github.com/LuppovDaniil/thymic_selection_motifs_database.
