# Supplementary material for: Comprehensive analysis of αβT-cell receptor repertoires reveals signatures of thymic selection
Source: Front Immunol. 2025 Sep 19;16:1605170. doi: 10.3389/fimmu.2025.1605170 (PMC12491325; doi:10.3389/fimmu.2025.1605170)
Supplement: Supplementary file 3 [file Table1.docx]

## Supplementary Figures and Tables


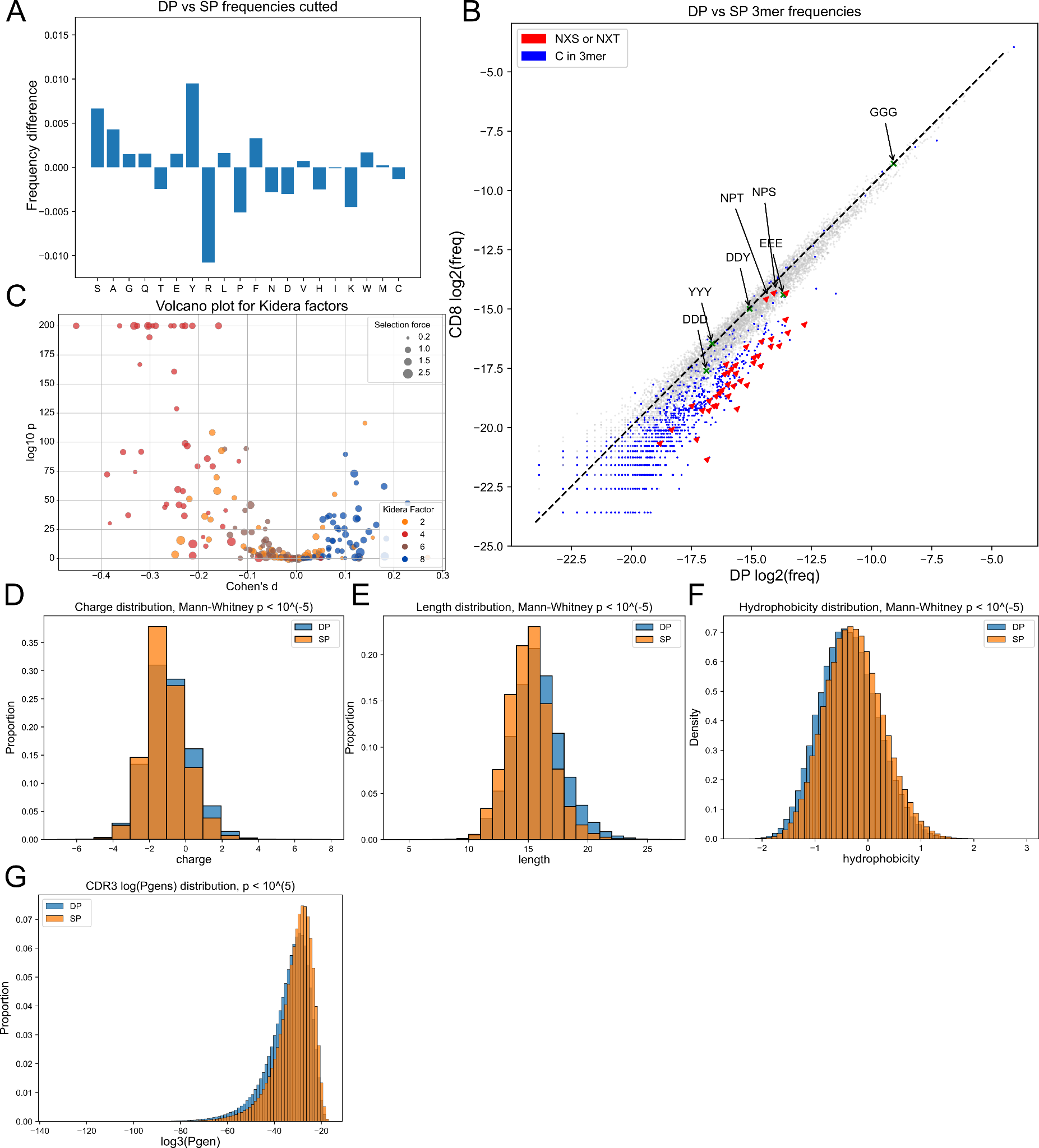


**Supplementary Figure 1. Comparing CDR3β sequences of double positive (DP) thymocytes and CD8+ single positive (SP) thymocytes. (A).** Amino acid frequency comparison between double positive and single positive thymocytes. **(B).** Comparing frequencies of k=3-mers between thymocyte subsets. Glycosylation sites (red) and Cys-containing 3-mers (blue) are negatively selected. **(C).** Volcano plot showing positive and negative selection P-values and effect size for four selected Kidera factors. Each point represents an independent calculation performed for a certain VJ pair. Results are similar to those observed in data generated using the VDJ rearrangement model, except for the effect for Kidera factor 6 that is smaller. **(D).** Negative shift of the charge distribution post-selection. **(E).** Shortening of CDR3 lengths post-selection. **(F).** Positive shift of the hydrophobicity distribution post-selection. **(G).** Generation probability (*Pgen*) distribution is higher for SP thymocytes compared to DP, in line with previous observation that variants with higher *Pgen* are more likely to pass selection.


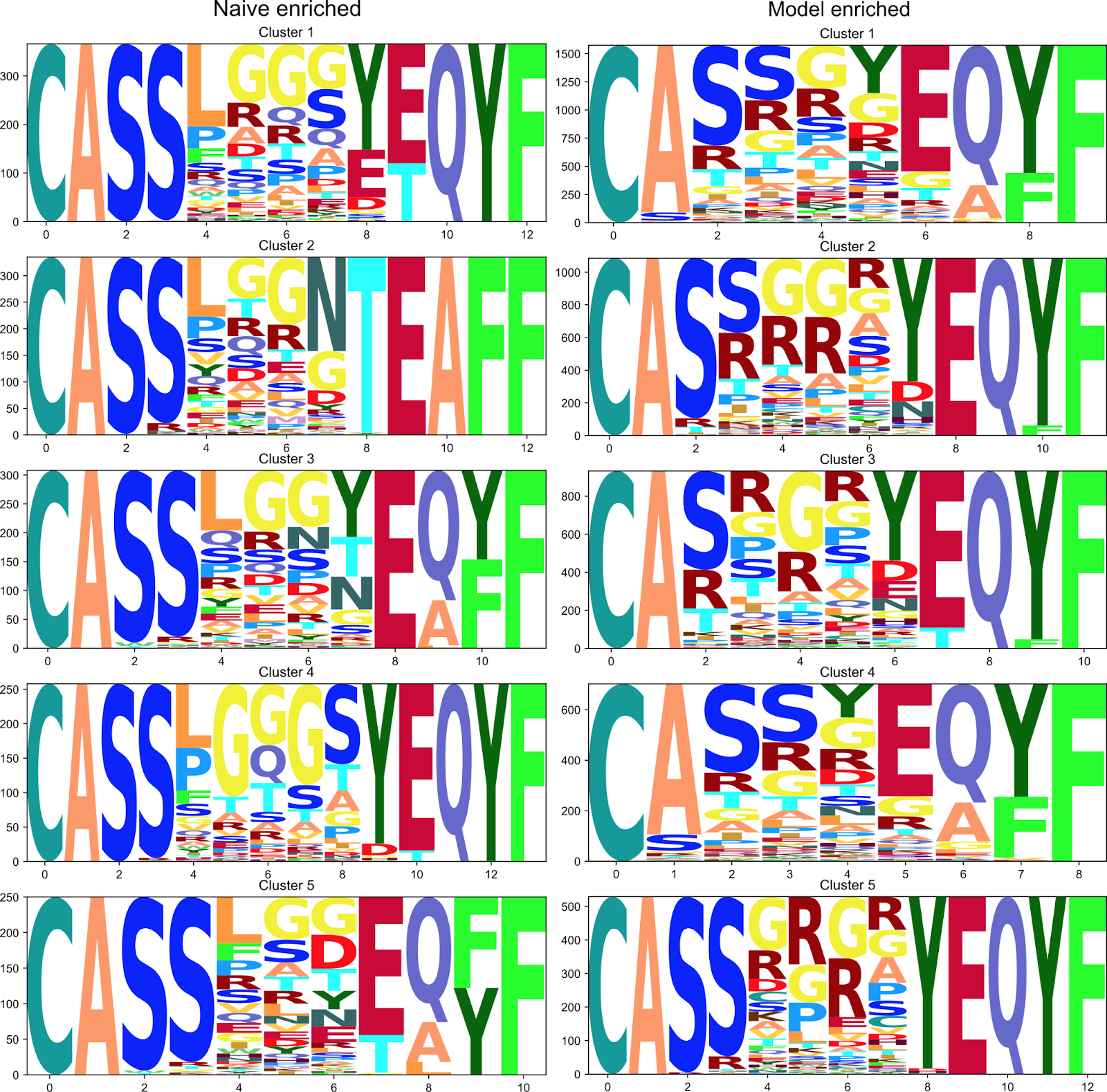


**Supplementary Figure 2.** Top 5 largest clusters of TCR-CDR3β sequences enriched in naive T-cell sample (enriched after the selection, left panel) and in a sample generated using VDJ rearrangement model- (depleted after the selection, right panel). Note that flexible poly-G motifs are favored by selection while Arg residues are negatively selected.


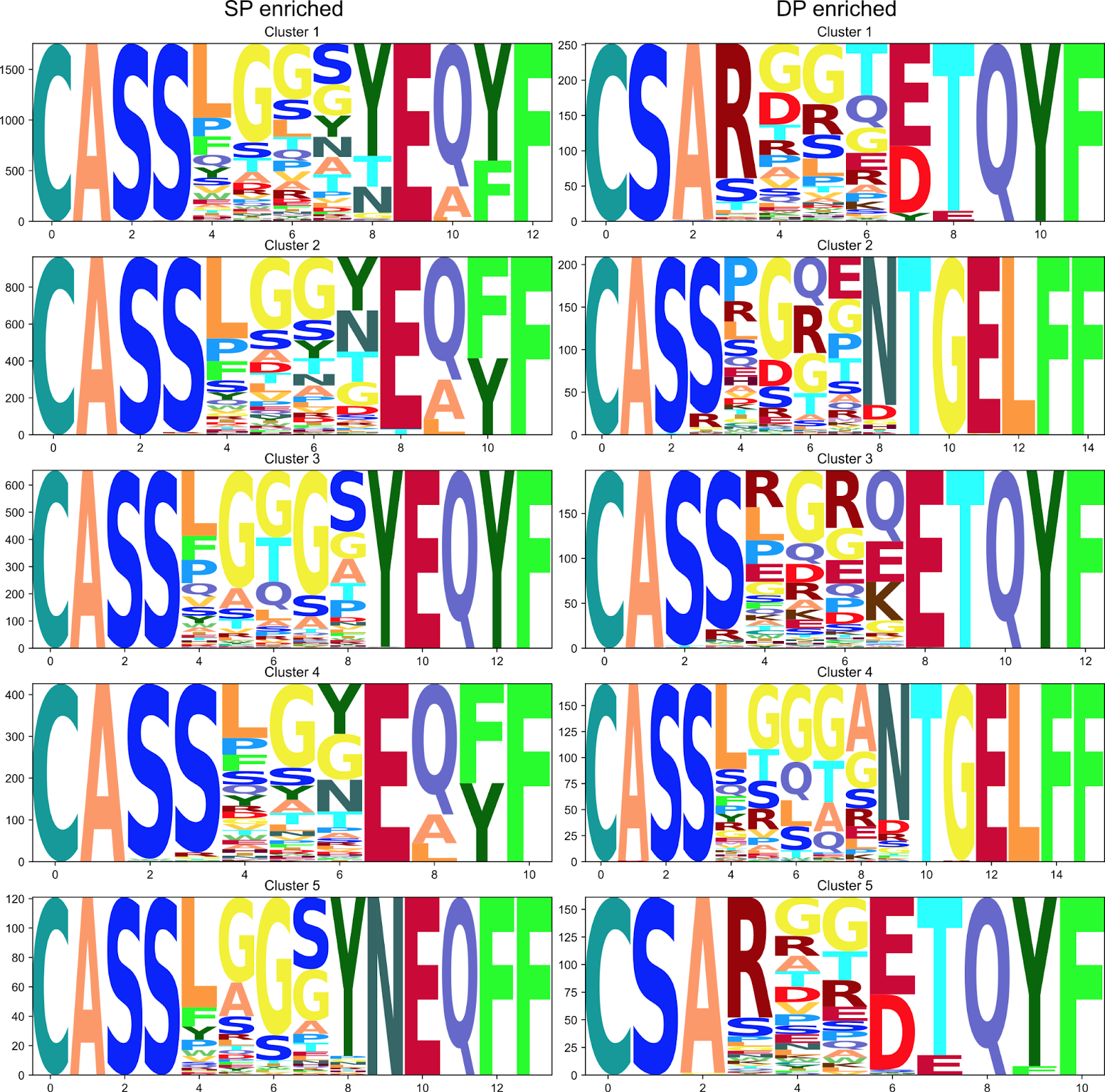


**Supplementary Figure 3.** Same as in **Supplementary Figure 2**, but comparing CD8+ single-positive (SP) as a proxy for positively selected TCR-CDR3β motifs and double-positive (DP) as a proxy for pre-selection thymocytes.


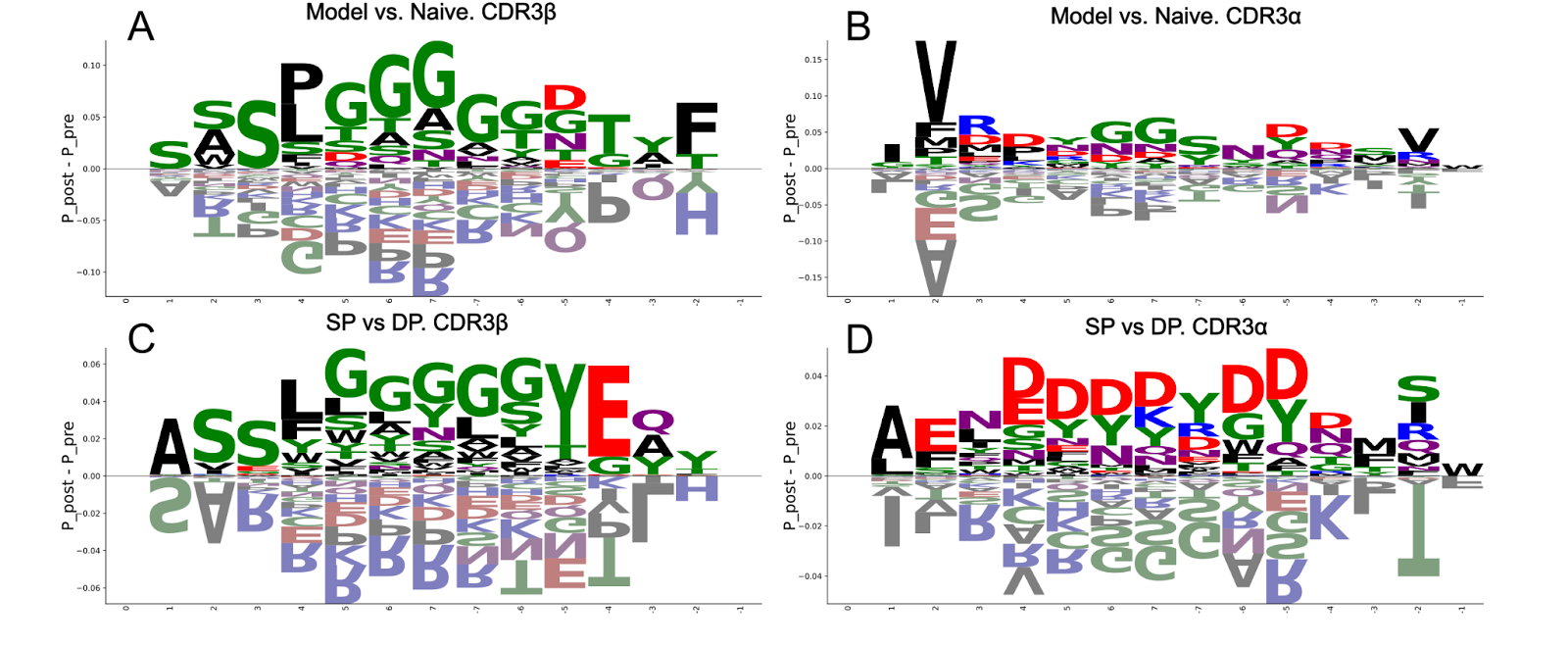


**Supplementary Figure 4.** Difference in marginal probabilities of amino acid occurrence learned by SoNNia thymic selection model visualized using sequence logos. VDJ rearrangement model vs naive cells and DP vs SP CDR3α and β are shown.


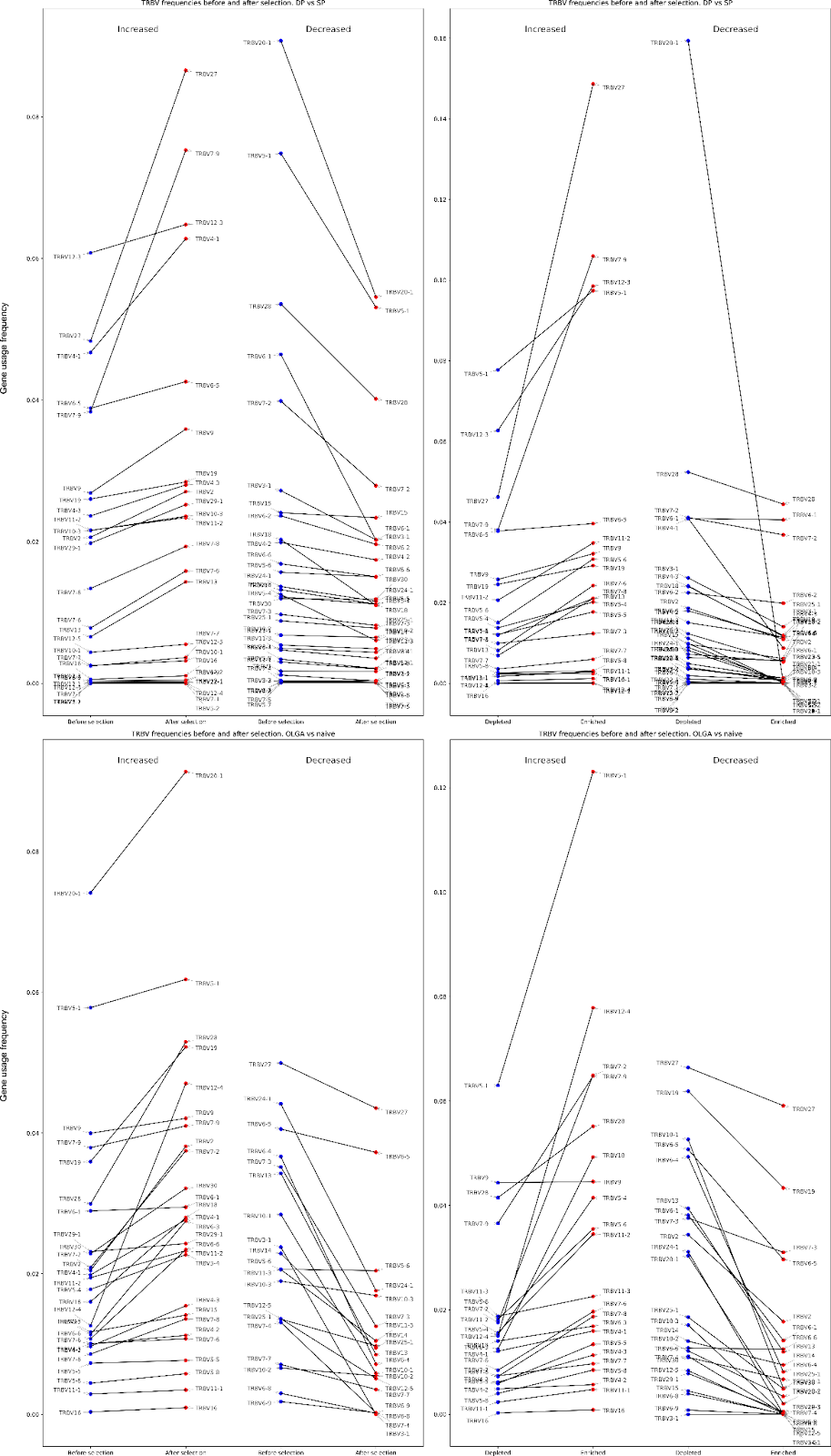


**
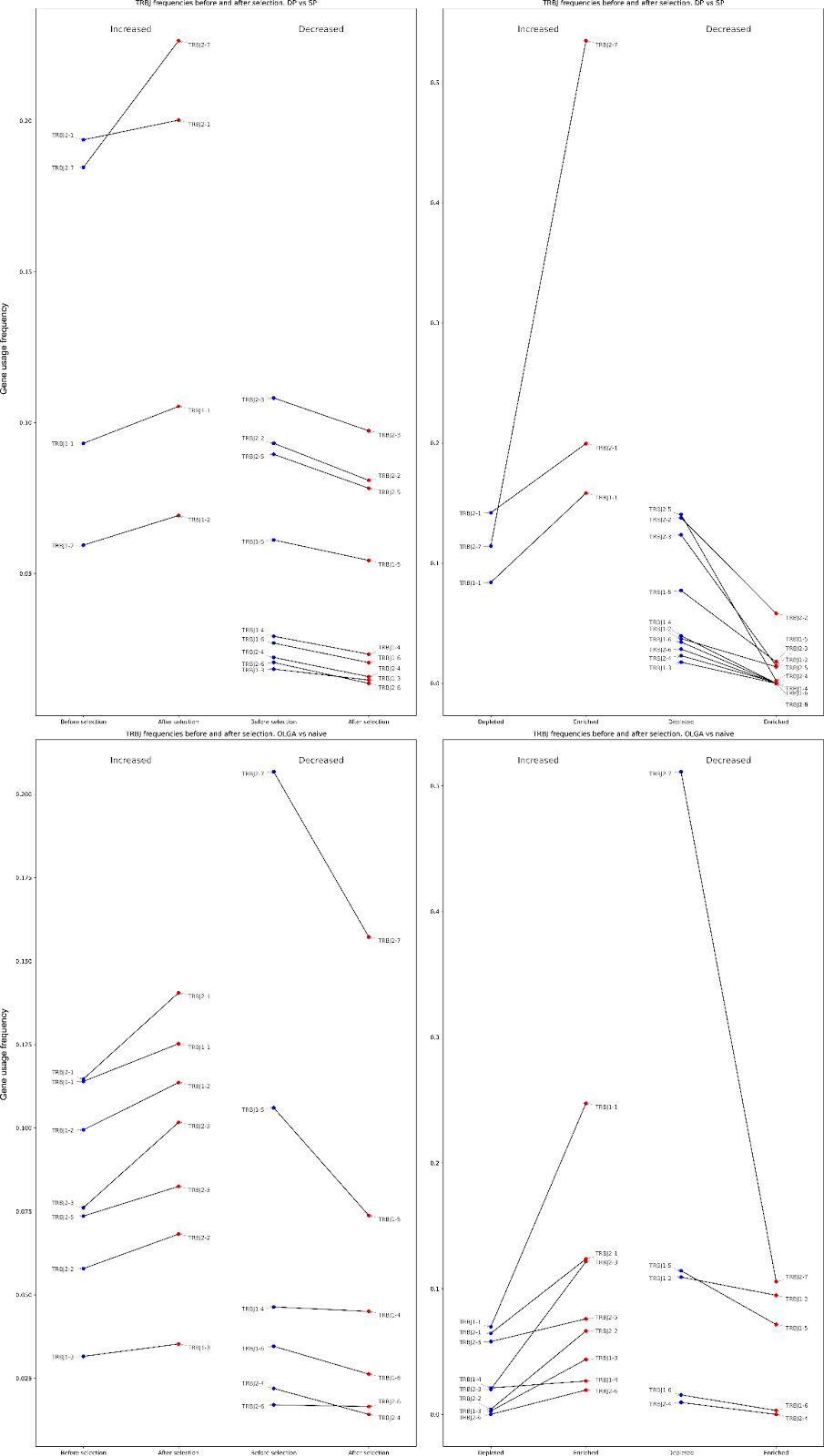
Supplementary Figure 5.** TCRβ gene Variable and Joining gene usage before and after selection. Analysis was carried out both for whole repertoires and for selected enriched and depleted after thymic selection TCRβ CDR3 clusters. This figure is also attached as a separate Supplementary file.


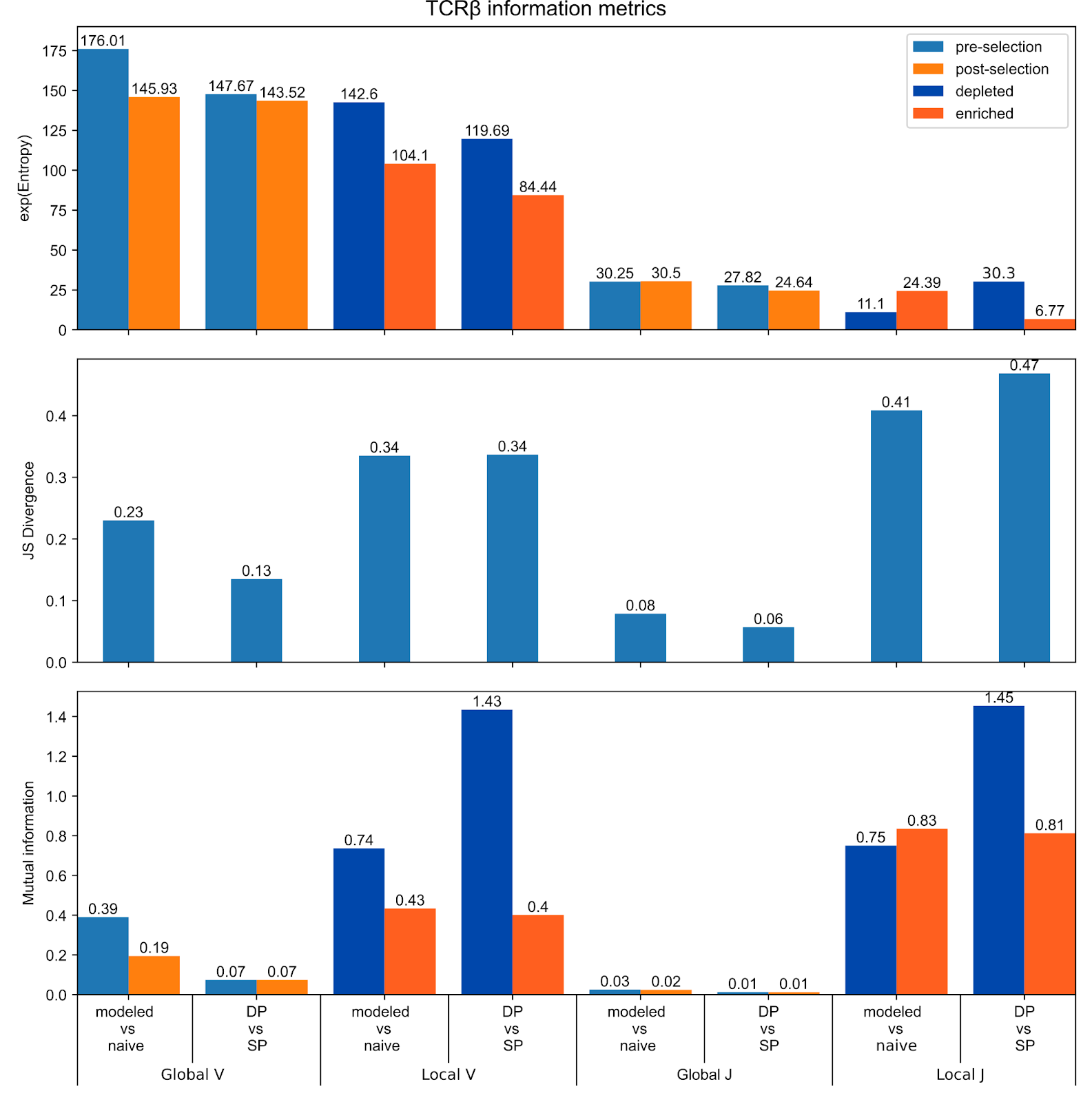


**Supplementary Figure 6.** TCRβ V and J gene usage information metrics. Analysis was carried out for a whole repertoire (Global) and for enriched pre-/post-selection TCRβ CDR3 clusters (Local).


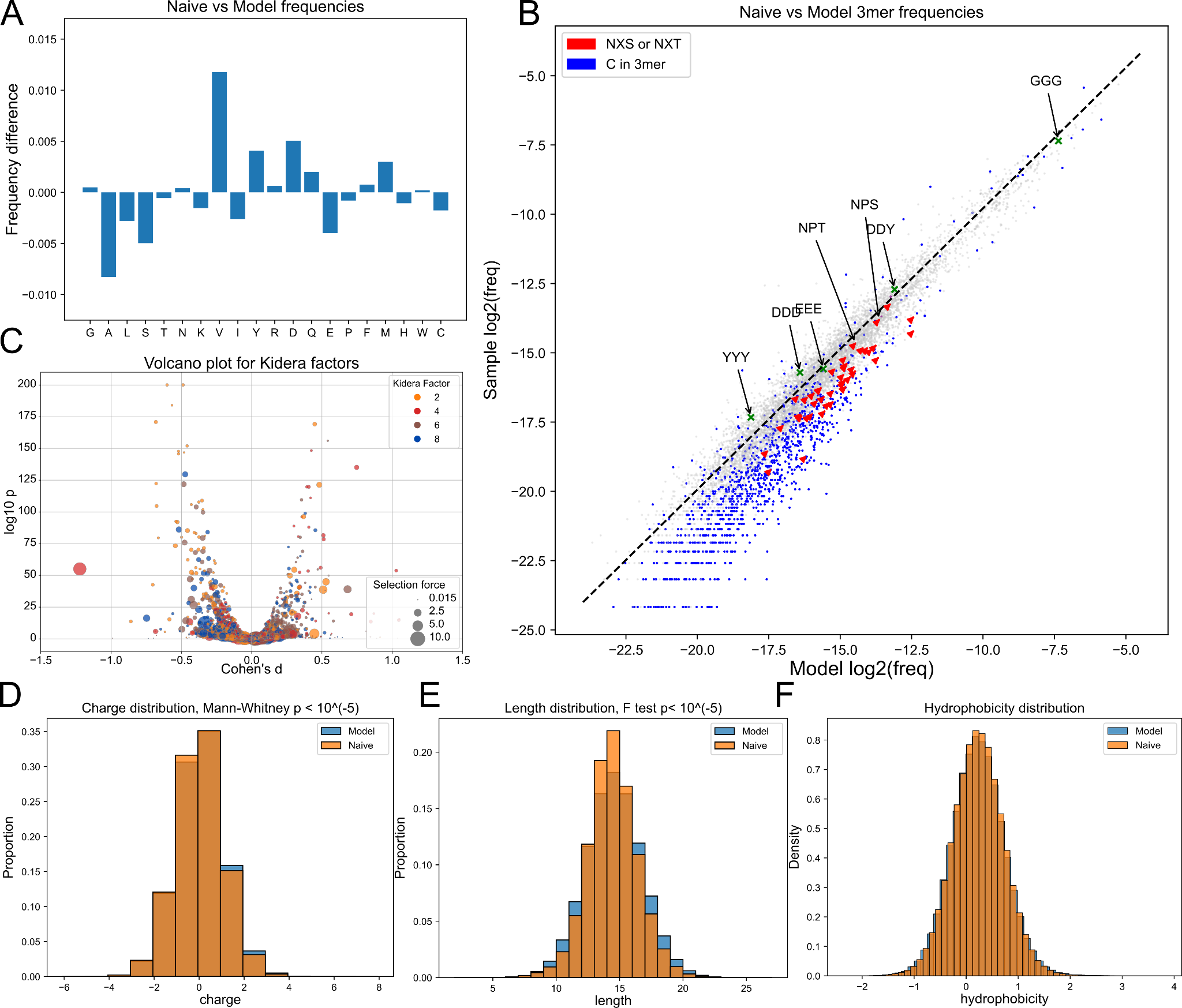


**Supplementary Figure 7.** Same as **Figure 1**, but for the TCRα chain. Model-generated CDR3α and naive CDR3α repertoire comparison. **(A).** Amino acid frequency comparison between repertoire generated with VDJ rearrangement model and naive T-cells. **(B).** Comparing frequencies of k=3-mers between model generated and naive subsets. Glycosylation sites (red) and Cys-containing 3-mers (blue) are negatively selected. **(C).** Volcano plot showing positive and negative selection P-values and effect size for four selected Kidera factors. Each point represents an independent calculation performed for a certain VJ pair. Direction of selection appears to be dependent on VJ-pair choice since there is no common direction for each Kidera factor selection. **(D).** Negative shift on charge distribution post-selection. **(E).** “Winsorizing” of CDR3 length distribution post-selection, extremely short and long CDR3s are removed during selection. **(F).** Hydrophobicity remains unchanged post-selection unlike CDR3β data.


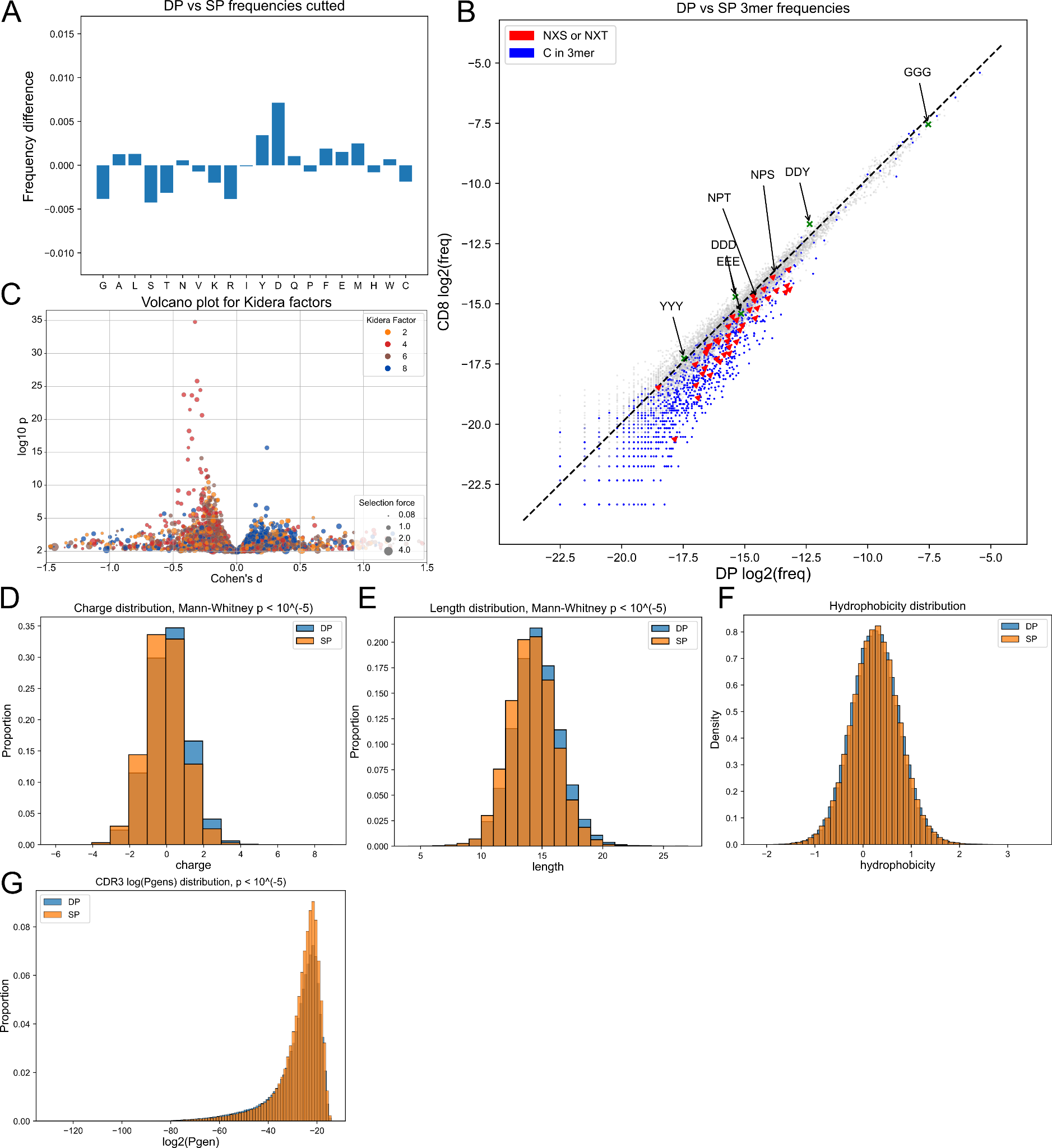


**Supplementary Figure 8.** Same as **Supplementary Figure 1**, but for the TCRα chain. Double positive (DP) thymocytes CDR3α and CD8 single positive (SP) thymocytes CDR3α repertoires comparison. **(A).** Amino acid frequency comparison between double positive and single positive thymocytes. **(B).** Comparing frequencies of k=3-mers between thymocyte subsets. Glycosylation sites (red) and Cys-containing 3-mers (blue) are negatively selected. **(C).** Volcano plot for Kidera factors affected by selection. Each point represents a VJ pair. Change appears to be dependent on VJ choice as it was observed on generated data. **(D).** Negative shift of the charge distribution post-selection. **(E).** Shortening of CDR3 lengths. **(F).** Hydrophobicity remains unchanged post-selection as it is in the generated data. **(G).** Generation probability (*Pgen*) distribution is higher for SP thymocytes compared to DP, in line with previous observation that variants with higher *Pgen* are more likely to pass selection.


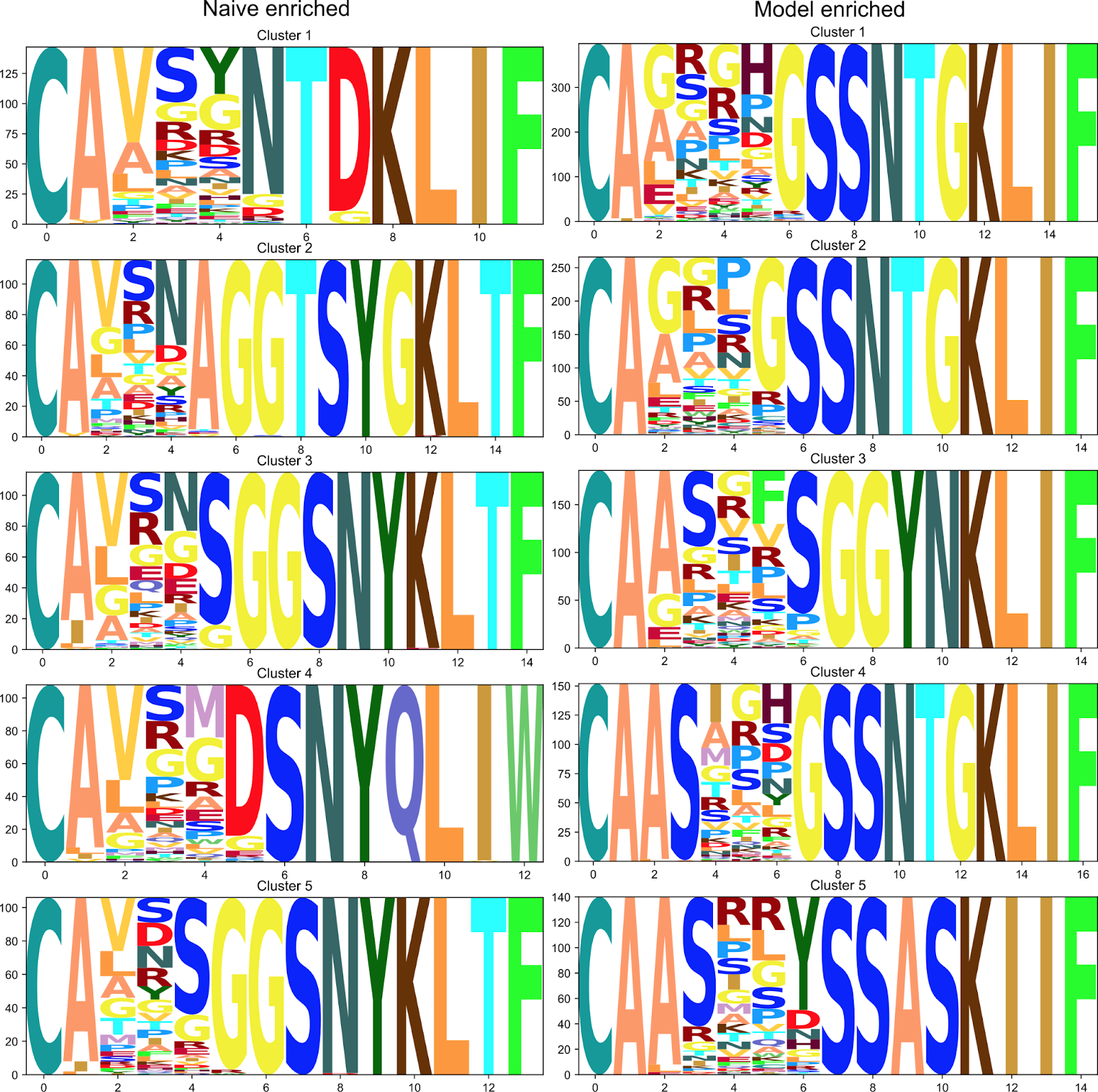


**Supplementary Figure 9.** Same as **Supplementary Figure 2** but comparing TCR-CDR3α clusters. In naive T-cells enriched clusters there are a lot of flexible poly-G motifs. In model-enriched clusters there are a lot of SS motifs.


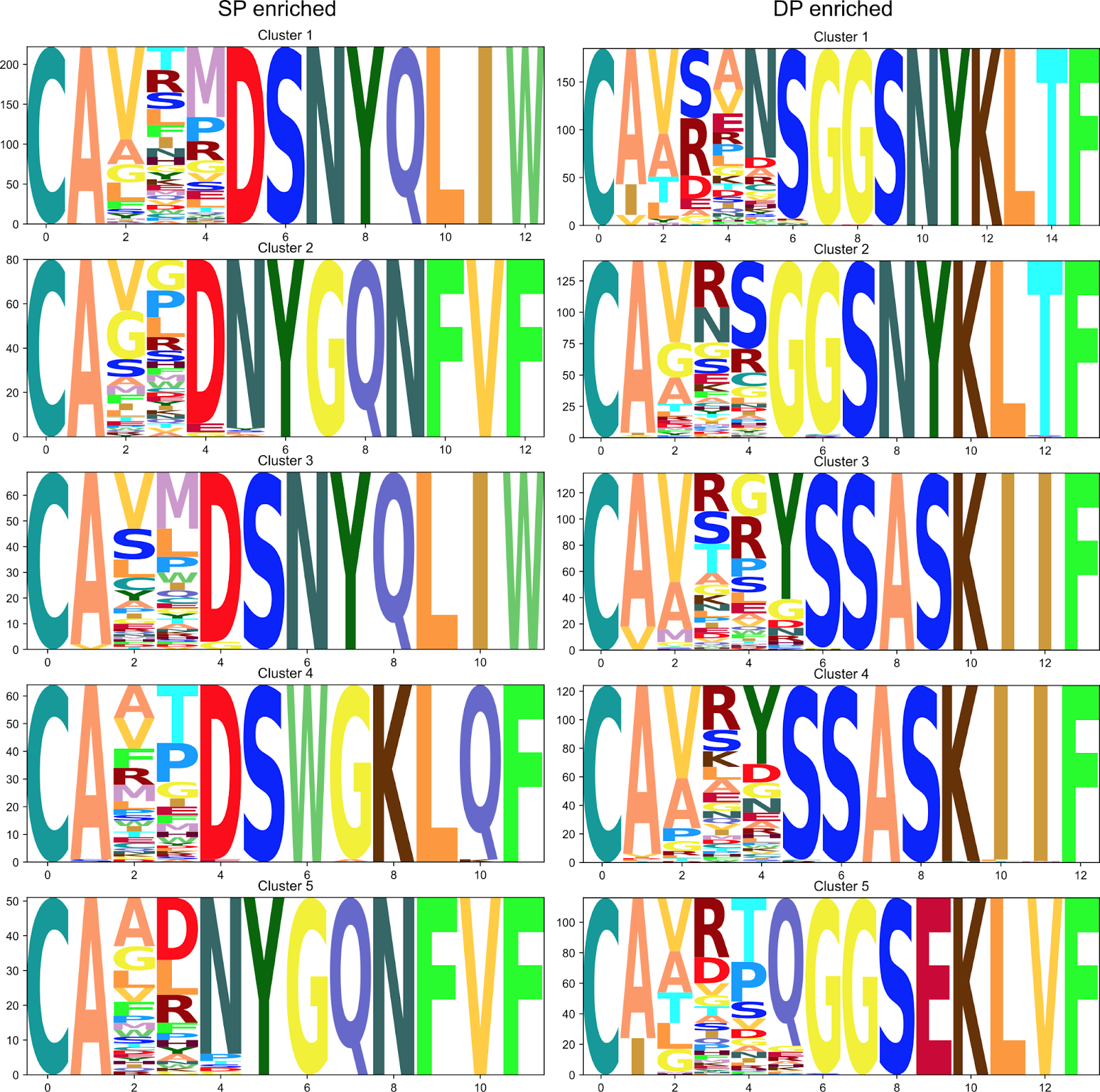


**Supplementary Figure 10.** Same as in **Supplementary Figure 2**, but comparing CD8+ single-positive (SP) as a proxy for positively selected TCR-CDR3α motifs and double-positive (DP) as a proxy for pre-selection thymocytes.


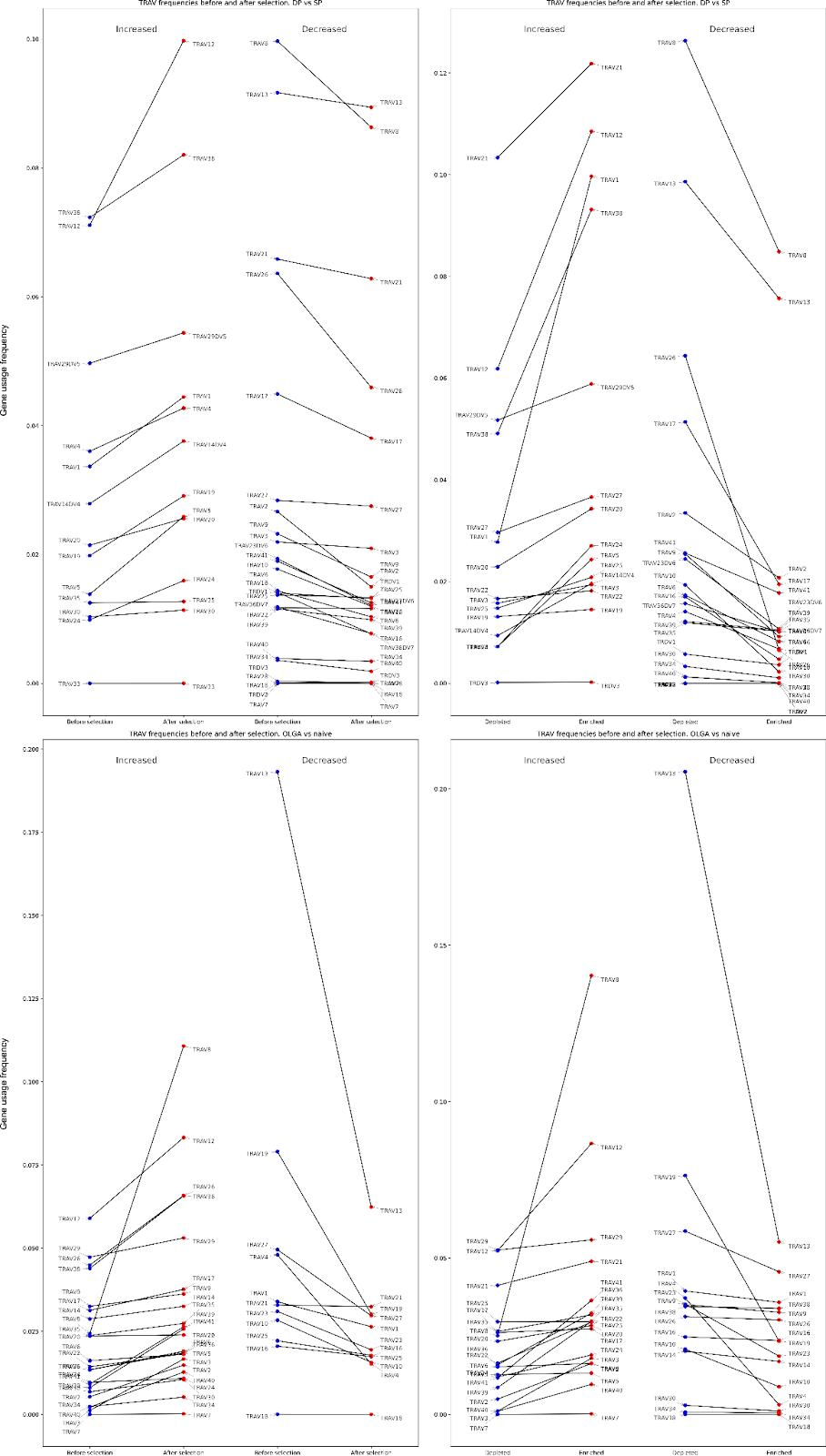


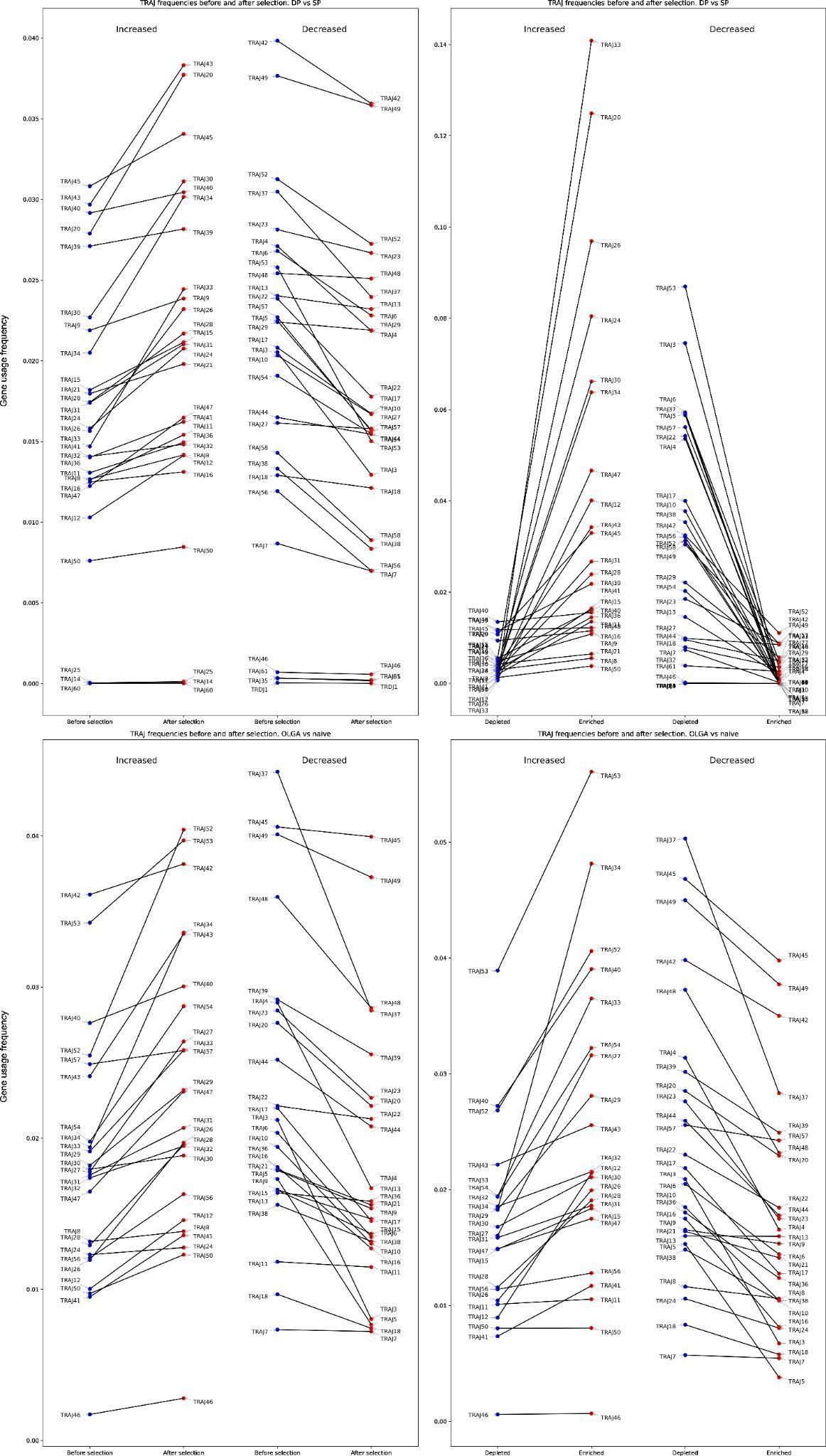


**Supplementary Figure 11.** TCRα V and J gene usage before and after selection. Analysis was carried out for entire TCR repertoires and for the enriched and depleted after thymic selection TCRα clusters. This figure is also attached as a separate Supplementary file.


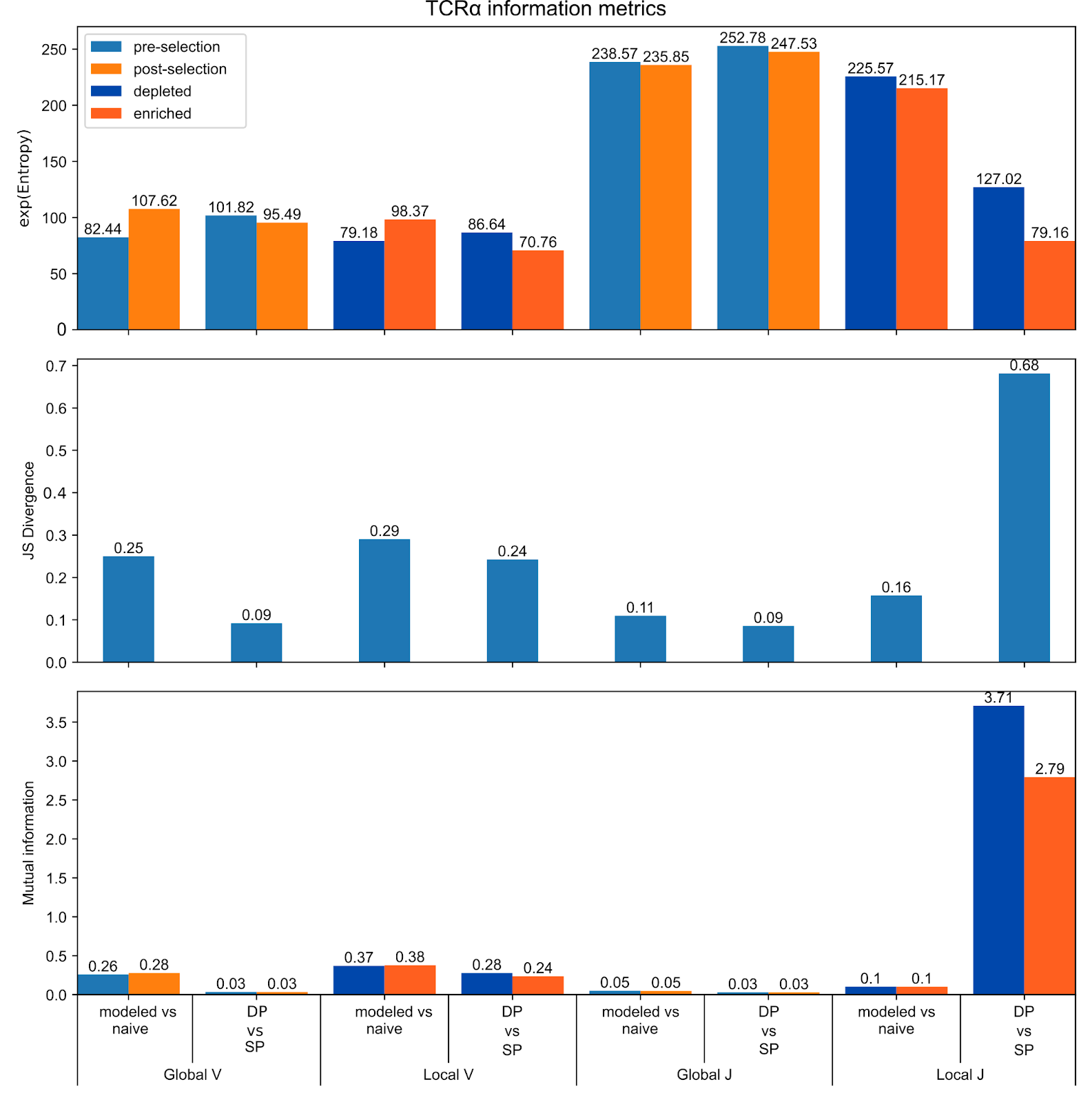


**Supplementary Figure 12.** TCRα V and J gene usage information metrics. Analysis was carried out for a whole repertoire (Global) and for enriched in pre-/post- selection repertoires (Local).

**Supplementary Table 1.** Metadata of datasets used in the study, number of detected clonotypes (unique VDJ junctions) and number of donors is reported.

| Dataset | Chain | # of clones analyzed | # of subjects | protocol |
| --- | --- | --- | --- | --- |
| Emerson*et al.* | β | 1147250 | 10 | Adaptive |
| Heikkilä *et al.* | α | 1582774 | 10 | Adaptive |
| Qi *et al.*, CD4+ | β | 1599217 | 9 | Custom |
| Qi *et al.*, CD8+ | β | 1346247 | 9 | Custom |
| Quiniou *et al.* | β | 1134755 | 14 | 5’ RACE, no UMI |
| Quiniou *et al.* | α | 902723 | 14 | 5’ RACE, no UMI |
| Pogorelyy *et al.* | β | 3678636 | 6, 2 replicas | 5’ RACE, with UMI |

**Supplementary Table 2.** HLA typing for 3 pairs of twins from Pogorelyy *et al.* study, class I and II alleles are reported.

|  | S1 and S2 | P1 and P2 | Q1 and Q2 |
| --- | --- | --- | --- |
| A | 02:01:01/03:01:01 | 02:01:01/02:01:01 | 02:01:01/02:01:01 |
| B | 38:01:01/07:02:01 | 51:01:01/07:02:01 | 13:02:01/44:02:01 |
| C | 07:02:01/12:03:01 | 07:02:01/14:02:01 | 05:01:01/06:02:01 |
| DQB1 | 06:03:01/06:02:01 | 03:01:01/04:02:01 | 02:02:01/06:03:01 |
| DRB1 | 13:01:01/15:01:01 | 08:03:02/08:01:03 | 07:01:01/13:01:01 |
| DRB3 | 1:01:02 | - | 1:01:02 |
| DRB4 | - | - | 1:03 |

###

### Supplementary Note 1

For additional CDR3 driven lineage commitment research we utilized a standard 10X Genomics dataset containing 20,000 PBMCs of a healthy donor (<https://www.10xgenomics.com/resources/datasets/20-k-human-pbm-cs-5-ht-v-2-0-2-high-6-1-0>) - “Dataset #1”.

We matched the enriched in SP thymocytes CDR3 sequences with CDR3 sequences from a Dataset #1 (Supplementary Figures 13**(A, B)**) and annotated cells, which carried matched CDR3s. Cells with CDR3β enriched in CD8+ thymocytes dataset were expectedly abundant mostly in CD8+ cluster (p < 0.05, Fisher exact test) (Supplementary Figures 13**(A, C)**). However, CD4+ cells also carried enriched TCRs, highlighting the partial stochasticity of lineage commitment in the thymus [(1)](https://www.zotero.org/google-docs/?ZZaphv).


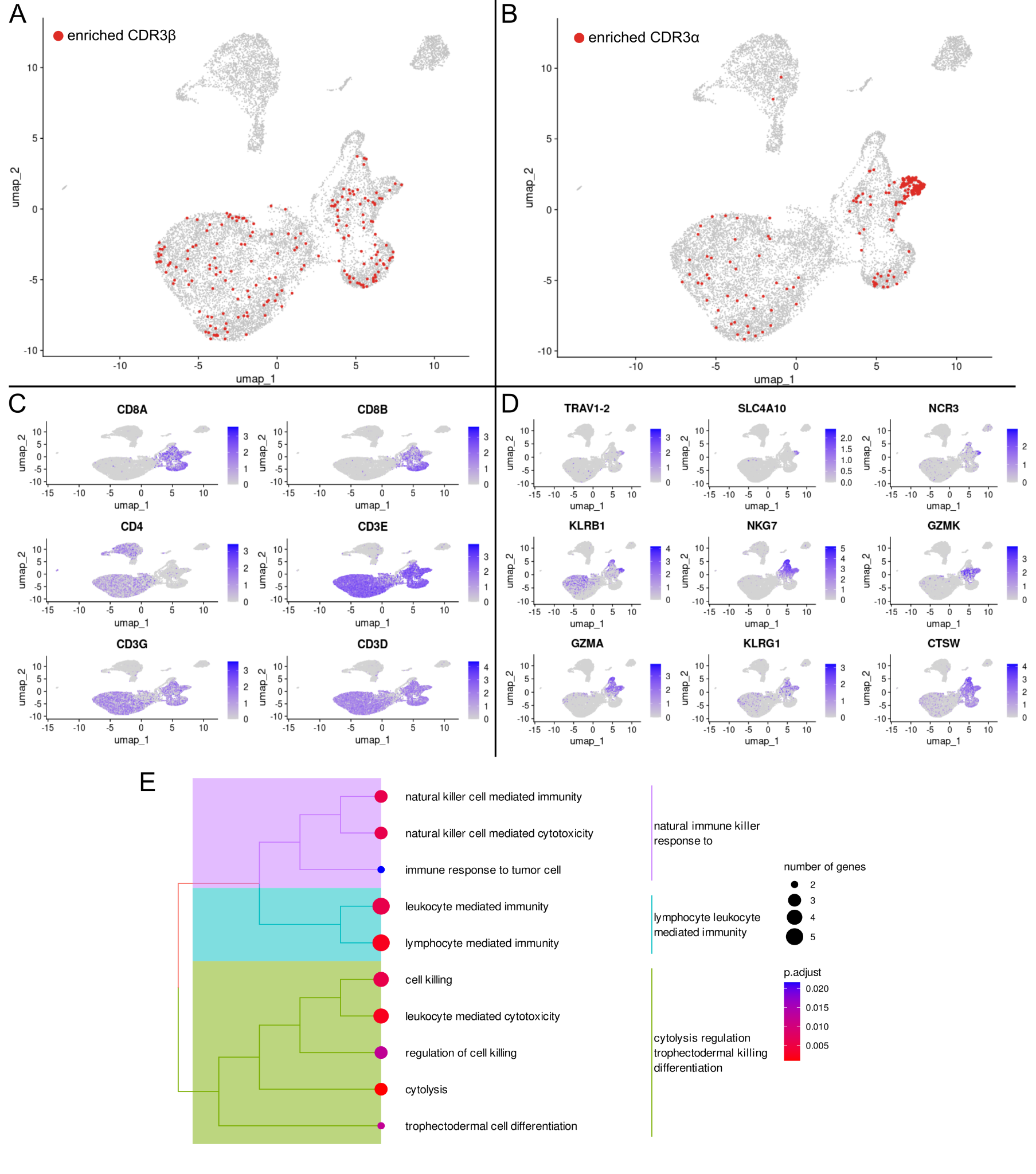


**Supplementary Figure 13.** Single-cell analysis of cells carrying enriched after thymic selection CDR3s for Dataset #1. **(A).** CDR3β enriched repertoires mapped to single-cell UMAP plot. **(B).** CDR3α enriched repertoires mapped to single-cell UMAP plot. **(C).** T-cell markers on a single-cell UMAP plot. Large proportion of the cells from supplementary figure 13A are in the CD8 rich area. **(D).** Top 9 differentially expressed genes in cells with CDR3 identical to those enriched in CDR3α CD8 dataset mapped to UMAP plot. TRAV1-2 and KLRB1, which were differentially expressed, are markers of MAIT cells. **(E).** Gene ontology (GO) terms enriched in top 20 differentially expressed genes of CDR3α group. They are similar to GO terms enriched in genes expressed by MAIT cells.

For clusters enriched in CDR3α SP sample we discovered a subset of cells densely located in a localized region of the UMAP plot (Supplementary Figure 13 **(B)**). All cells which carried enriched TCRs were taken into differential expression analysis. Visualization of top 9 differentially expressed genes is shown on Supplementary Figure 13**(D)**. Genes such as TRAV1-2 and KLRB1, which were differentially expressed in our group identify a distinct subset of lymphocytes known as mucosal-associated invariant T cells (MAIT cells) [(2)](https://www.zotero.org/google-docs/?8xGfb5).

We performed above analysis using 2 other datasets:

<https://www.10xgenomics.com/resources/datasets/human-pbmc-from-a-healthy-donor-10-k-cells-multi-v-2-2-standard-5-0-0> - “Dataset #2”

<https://www.10xgenomics.com/resources/datasets/10k-human-pbmcs-5-v2-0-chromium-x-without-intronic-reads-2-standard> - “Dataset #3”

The results for both additional Datasets were identical to those obtained above. Enriched CDR3β tend to be carried by cells with CD8+ phenotype while enriched CDR3α were mostly associated with MAIT cells.


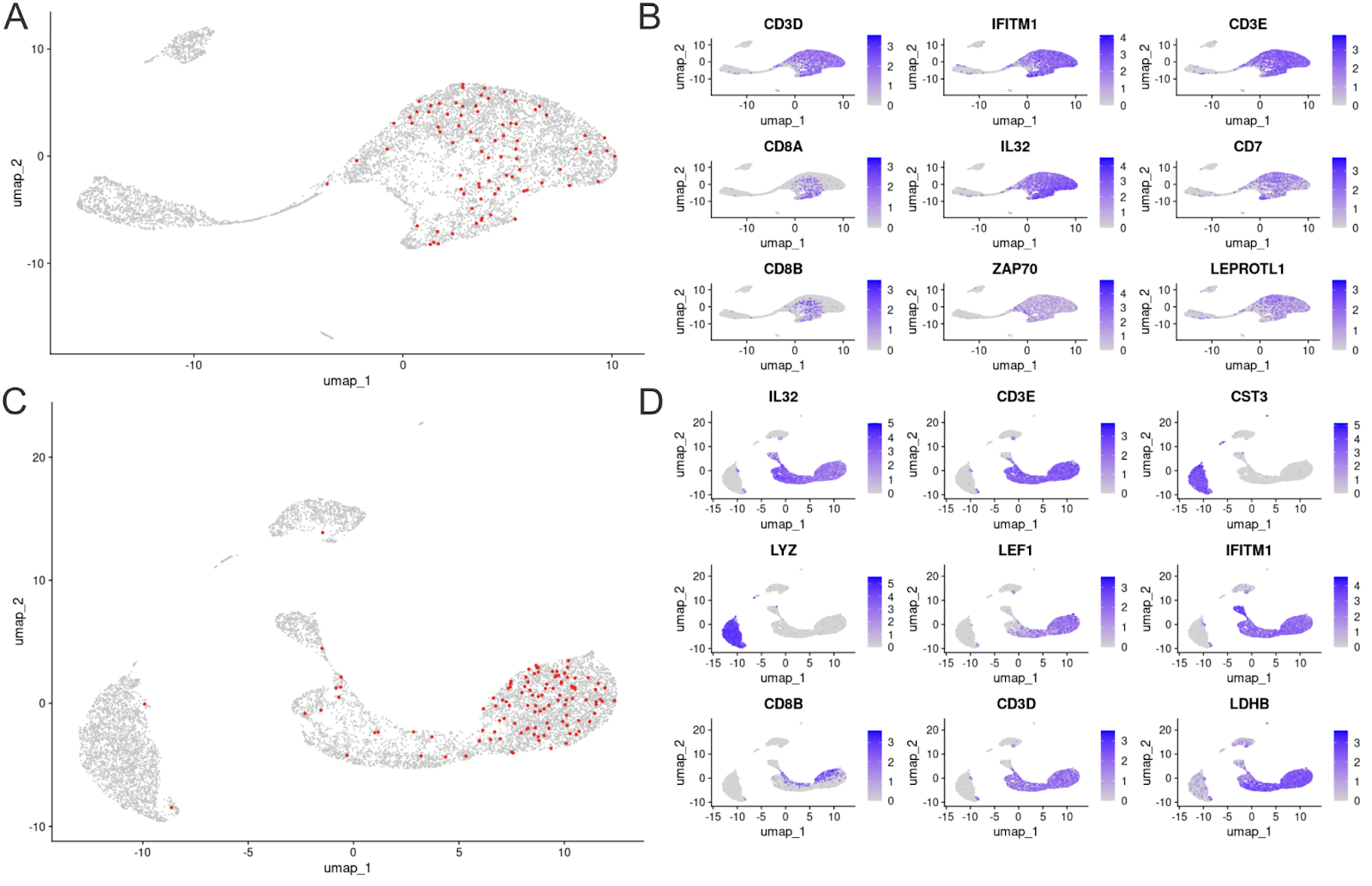


**Supplementary Figure 14. Single cell analysis of enriched after the selection CDR3β in 2 additional datasets.** **(A).** UMAP representation of Dataset #2 . **(B).** Top 9 differentially expressed genes among cells carrying enriched CDR3β in the Dataset #2. **(C).** Enriched CDR3β in UMAP representation of Dataset #3. **(D).** Top 9 differentially expressed genes, either over- or underexpressed, among cells carrying enriched CDR3β in the Dataset #3.


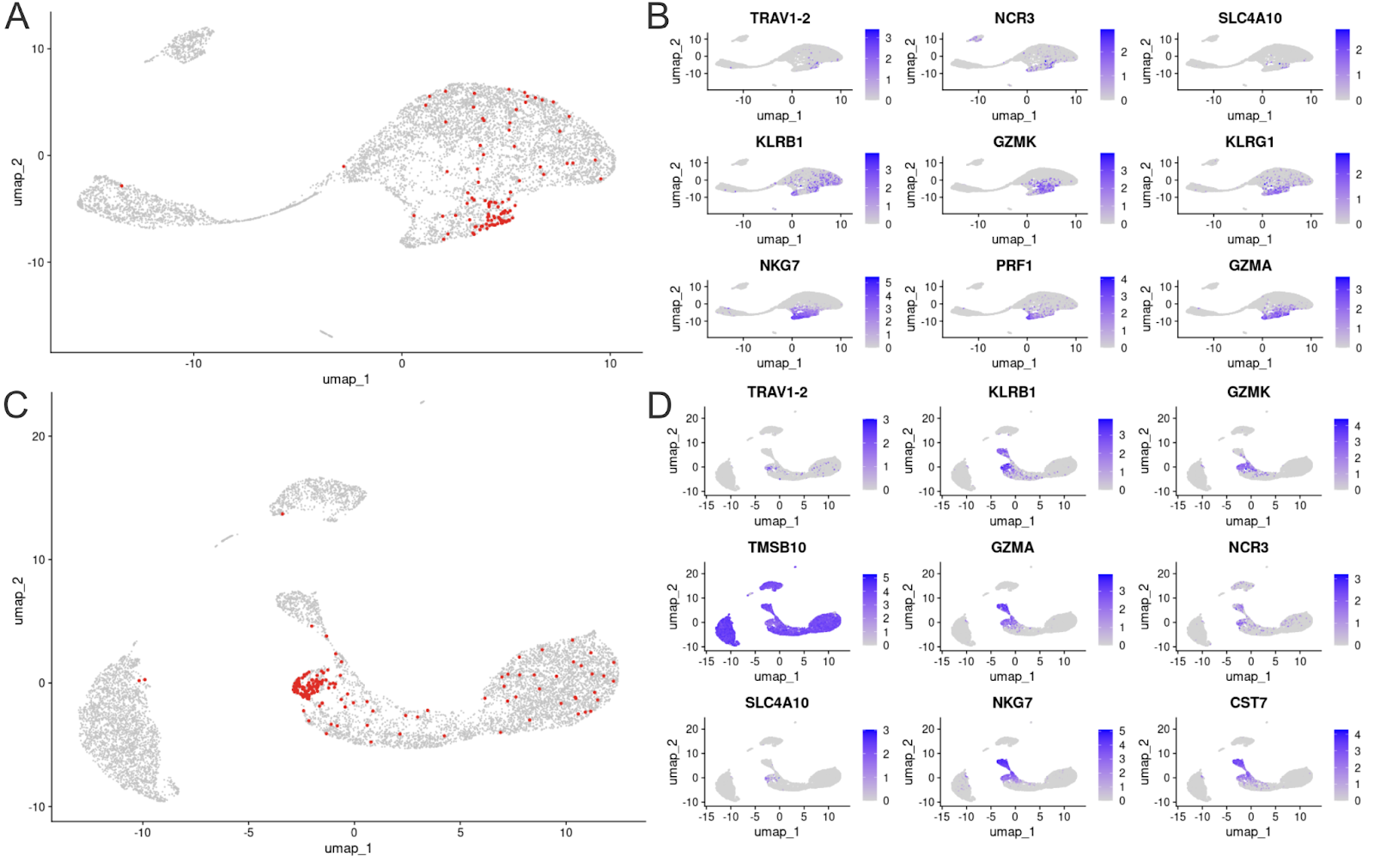


**Supplementary Figure 15.** Single-cell analysis of enriched CDR3α in 2 additional datasets. **(A).** CDR3α enriched repertoires mapped to single-cell UMAP plot of Dataset #2. **(B).** Top 9 differentially expressed genes among cells carrying enriched CDR3α in the Dataset #2. **(C).** CDR3α enriched repertoires mapped to single-cell UMAP plot of Dataset #3. **(D).** Top 9 differentially expressed genes among cells carrying enriched CDR3α in the Dataset #3.

For the above single cell data analysis we utilized the Seurat R package (version 4.9.9.9058) [(3)](https://www.zotero.org/google-docs/?vEMw0O). Data preparation, dimensionality reduction and UMAP visualization were carried out according to commonly used guidelines for 10X data: (https://satijalab.org/seurat/articles/pbmc3k_tutorial.html). Differential expression analysis for T-cells carrying TCR sequences of interest was performed using MAST algorithm [(4)](https://www.zotero.org/google-docs/?d9er4v) implemented in Seurat package. Gene Ontology (GO) analysis was performed in R using clusterProfiler R BioConductor package (version 4.8.2)[(5)](https://www.zotero.org/google-docs/?ECxIEk) .

### Supplementary Note 2

We reproduced the results of “HLA allele affects the selection” section using two additional datasets of twin TCR repertoires reported in [(6)](https://www.zotero.org/google-docs/?gauDfM) (dataset#2) and [(7)](https://www.zotero.org/google-docs/?SyEZxA) (dataset#3).

In dataset#2, the number of TCR clonotypes (unique VDJ junctions) in samples from each twin varied from 22k to 171k. We calculated Jensen–Shannon divergence for them in the same manner as for the dataset reported in the main text. This time the clustering for relative twins was much less pronounced. It is especially notable for sample a1, from which the number of clonotypes was smallest in the dataset (Supplementary Figure 16, Supplementary table 3). Thus, we speculate that lack of clustering may be due to insufficient sample size of data being analyzed.


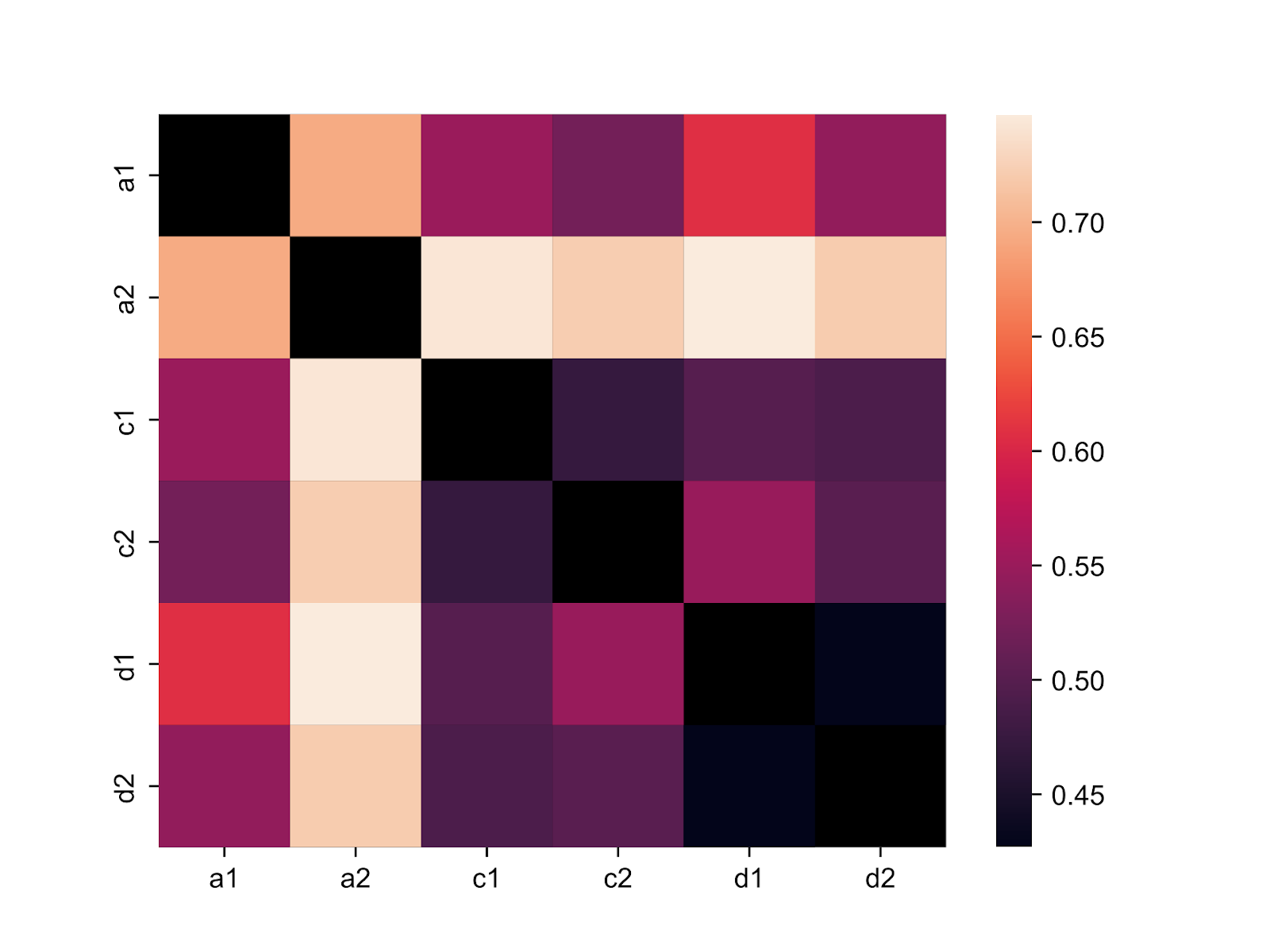


**Supplementary Figure 16.** Jensen-Shannon divergence for frequencies of positively-selected (enriched against a background sample produced using VDJ rearrangement model) CDR3β clusters in additional twin dataset (dataset #2).

**Supplementary Table 3.** Sample sizes of the additional twin dataset (dataset#2).

| Sample | TCRα clonotypes | TCRβ clonotypes |
| --- | --- | --- |
| a1 | 39378 | 53997 |
| a2 | 19134 | 22430 |
| c1 | 129837 | 171502 |
| c2 | 69964 | 78934 |
| d1 | 71951 | 91228 |
| d2 | 75272 | 166666 |

We then decided to check whether or not sample size affects clustering and what sample size is required for clustering to appear. To do so, we took the initial twin’s dataset (analyzed in a Result section “Twins CDR3 clusterization”) and then gradually reduced its sample size. Five replicas of sample size reduction were conducted. Then the mean Jensen-Shannon divergence was calculated for related twins and for unrelated twins along with confidence intervals.


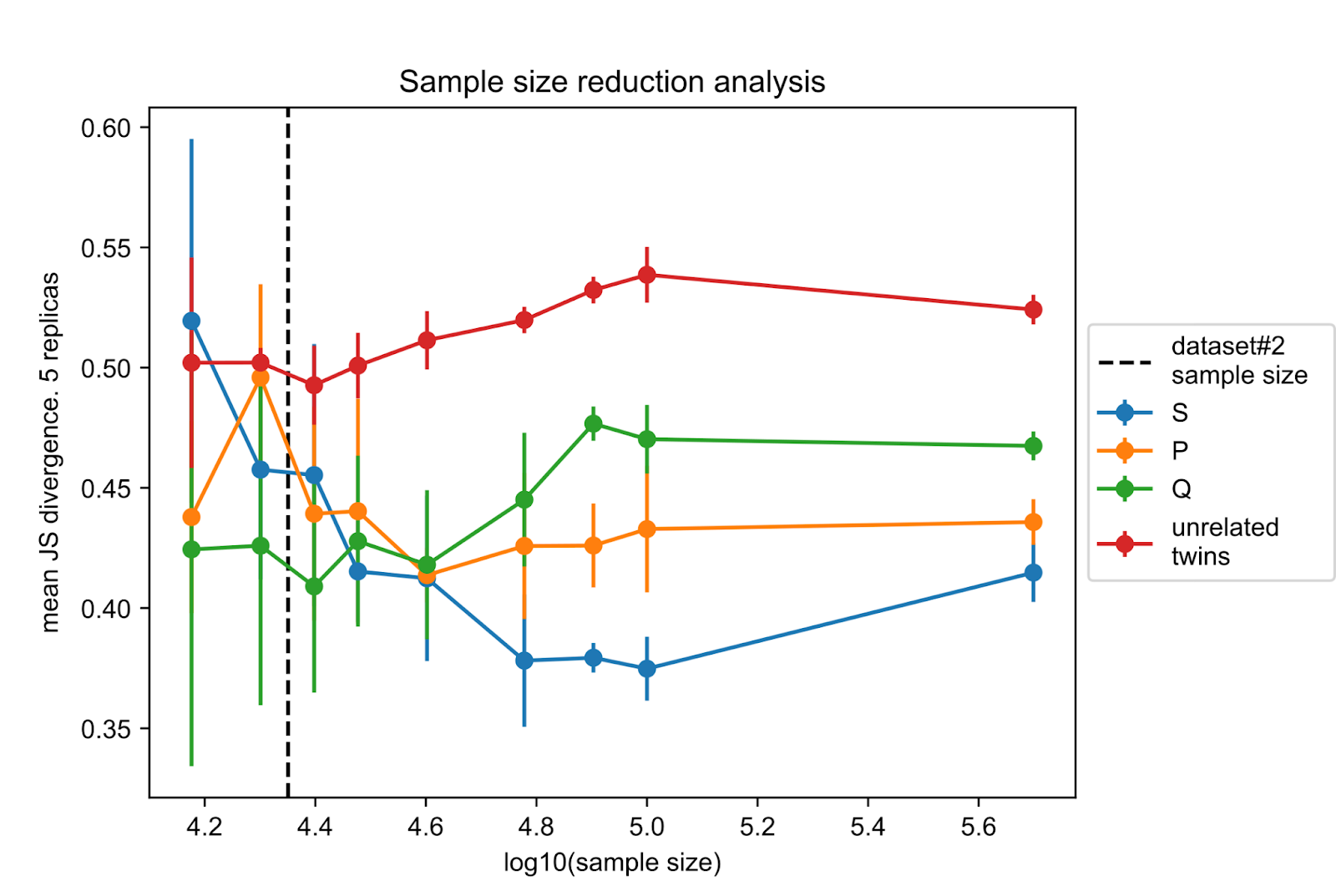


**Supplementary Figure 17.** Sample size affects the ability to distinguish relative and non-relative twins. Each point represents Jensen-Shannon divergence between relative twin pairs S, P, and Q or between unrelated twins averaged for five sample size reduction replicas. Error bars show confidence intervals calculated for 5 sample size reduction replicas. Dashed line represents the sample size of datasets#2.

Apparently, for successful HLA allele effect detection, at least 40k TCR clones from each sample are needed (Supplementary Figure 17). From this sample size, confidence intervals of relative twins no longer intersect with confidence intervals of unrelated twins. The sample size of datasets#2 (22k) is smaller than this value; thus, we can suggest that a modest sample size is the main reason why we failed to detect relative twins clustering in dataset#2.

Additionally, we ran clustering for motif frequences in dataset#3, which has CD4+ CDR3β from two pairs of twins. This dataset spans three subsets of T-cells for each sample: T-regulatory cells (Treg), recent thymic emigrants (RTEs) and all other cells. However, since the sample size of each subset was too small to detect clustering, we combined them together. (**Supplementary Table 4**).

We indeed detected the same effect as in the first dataset (Supplementary figure 18). However the effect on model enriched (depleted) TCR was not detected (Supplementary figure 19). It is still an open question whether this is due to not-sufficient sample size or due to absence of HLA allele effect on TCRs which did not pass through the selection in case of CD4+ cells.

**Supplementary Table 4.** Sample sizes of twins dataset#3 in terms of unique TCR beta sequences.

|  | Treg | RTE | N_nonRTE | Total |
| --- | --- | --- | --- | --- |
| J_1 | 27556 | 16765 | 72080 | 116401 |
| J_2 | 46922 | 38389 | 44957 | 130268 |
| C_1 | 33779 | 56979 | 38385 | 129143 |
| C_2 | 24927 | 64670 | 56000 | 145597 |


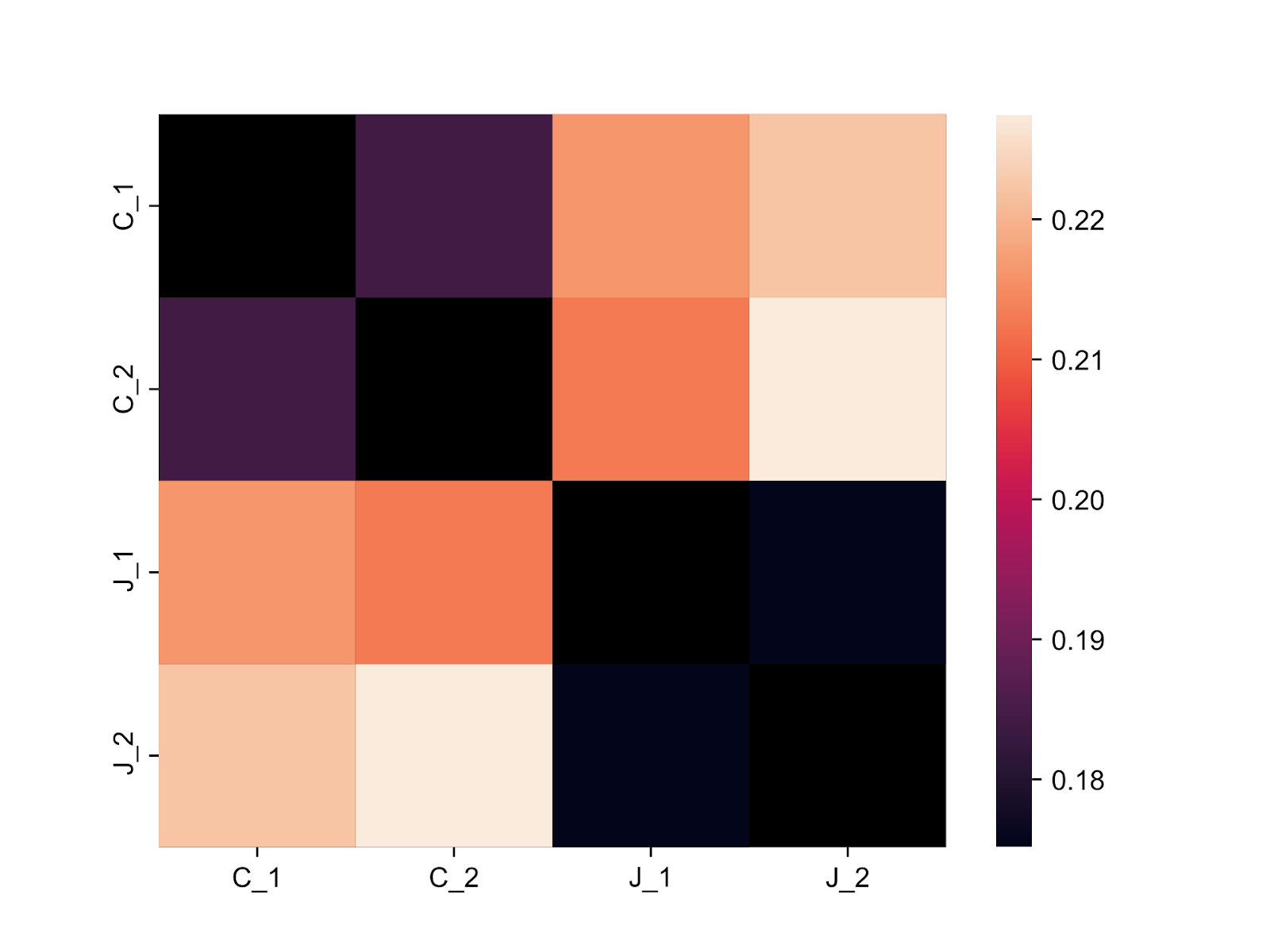


**Supplementary Figure 18.** Jensen-Shannon divergence for CDR3β clusters in dataset#3 enriched (positively selected) against a background dataset of TCRs generated using VDJ rearrangement model.


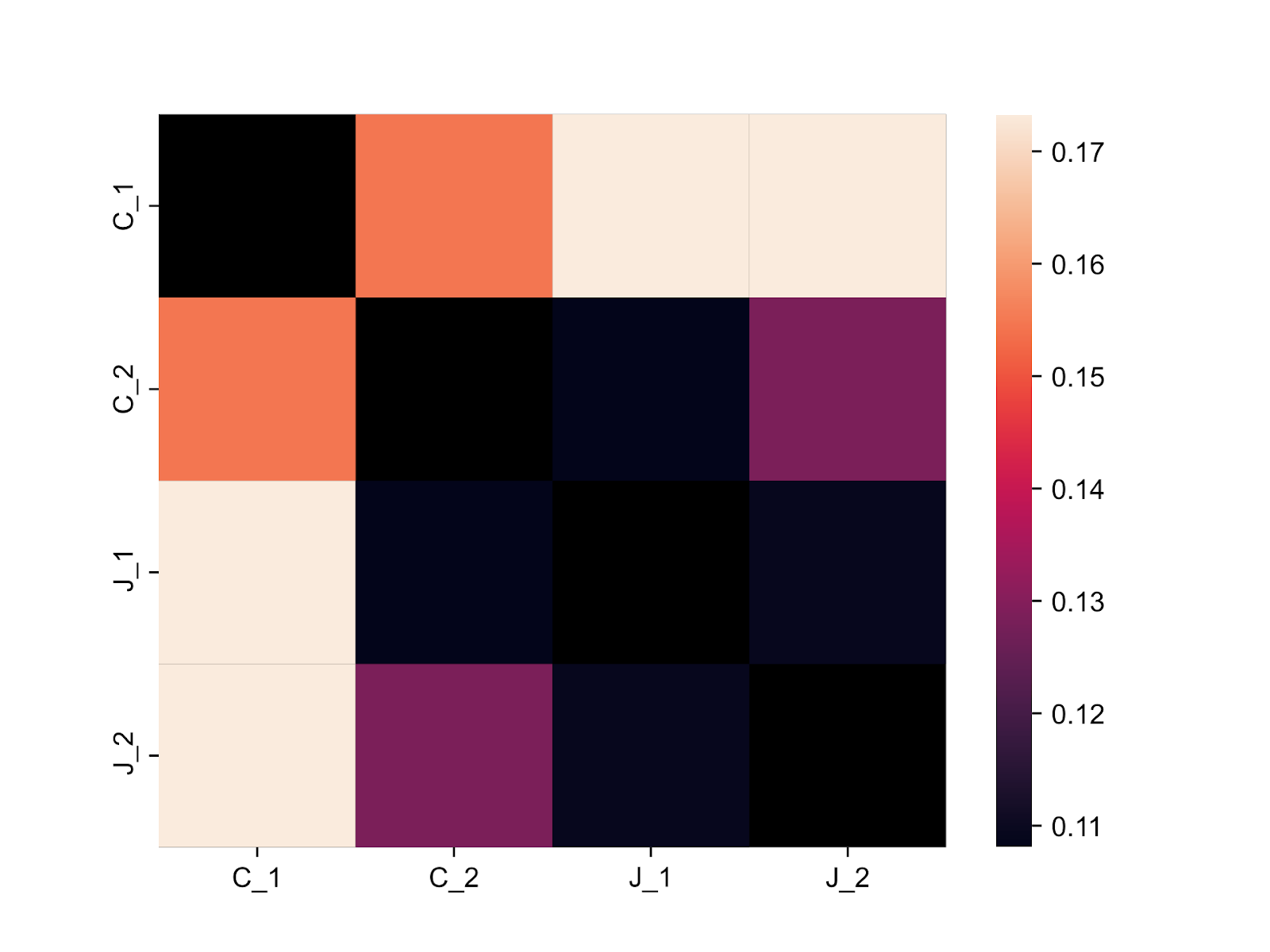


**Supplementary Figure 19.** Jensen-Shannon divergence for CDR3β clusters in dataset#3 depleted (negatively selected) against a background dataset of TCRs generated using VDJ rearrangement model.

## References

[1. Yates A. Theories and Quantification of Thymic Selection. *Frontiers in Immunology* (2014) 5: https://www.frontiersin.org/articles/10.3389/fimmu.2014.00013 [Accessed December 26, 2023]](https://www.zotero.org/google-docs/?rFrykc)

[2. Godfrey DI, Koay H-F, McCluskey J, Gherardin NA. The biology and functional importance of MAIT cells. *Nat Immunol* (2019) 20:1110–1128. doi: 10.1038/s41590-019-0444-8](https://www.zotero.org/google-docs/?rFrykc)

[3. Hao Y, Hao S, Andersen-Nissen E, Mauck WM, Zheng S, Butler A, Lee MJ, Wilk AJ, Darby C, Zager M, et al. Integrated analysis of multimodal single-cell data. *Cell* (2021) 184:3573-3587.e29. doi: 10.1016/j.cell.2021.04.048](https://www.zotero.org/google-docs/?rFrykc)

[4. Finak G, McDavid A, Yajima M, Deng J, Gersuk V, Shalek AK, Slichter CK, Miller HW, McElrath MJ, Prlic M, et al. MAST: a flexible statistical framework for assessing transcriptional changes and characterizing heterogeneity in single-cell RNA sequencing data. *Genome Biology* (2015) 16:278. doi: 10.1186/s13059-015-0844-5](https://www.zotero.org/google-docs/?rFrykc)

[5. Wu T, Hu E, Xu S, Chen M, Guo P, Dai Z, Feng T, Zhou L, Tang W, Zhan L, et al. clusterProfiler 4.0: A universal enrichment tool for interpreting omics data. *Innovation* (2021) 2: doi: 10.1016/j.xinn.2021.100141](https://www.zotero.org/google-docs/?rFrykc)

[6. Zvyagin IV, Pogorelyy MV, Ivanova ME, Komech EA, Shugay M, Bolotin DA, Shelenkov AA, Kurnosov AA, Staroverov DB, Chudakov DM, et al. Distinctive properties of identical twins’ TCR repertoires revealed by high-throughput sequencing. *Proceedings of the National Academy of Sciences* (2014) 111:5980–5985. doi: 10.1073/pnas.1319389111](https://www.zotero.org/google-docs/?rFrykc)

[7. Kasatskaya SA, Ladell K, Egorov ES, Miners KL, Davydov AN, Metsger M, Staroverov DB, Matveyshina EK, Shagina IA, Mamedov IZ, et al. Functionally specialized human CD4+ T-cell subsets express physicochemically distinct TCRs. *eLife* 9:e57063. doi: 10.7554/eLife.57063](https://www.zotero.org/google-docs/?rFrykc)
